# The effectiveness of testing, vaccinations and contact restrictions for containing the CoViD-19 pandemic

**DOI:** 10.1038/s41598-022-12015-9

**Published:** 2022-05-16

**Authors:** Janoś Gabler, Tobias Raabe, Klara Röhrl, Hans-Martin von Gaudecker

**Affiliations:** 1 Bonn Graduate School of Economics, 53113 Bonn, Germany; 2 IZA Institute of Labor Economics, 53113 Bonn, Germany; 3 Private sector, Hamburg, Germany; 4 Rheinische Friedrich-Wilhelms-Universität Bonn, 53113 Bonn, Germany

**Keywords:** Computational models, Viral infection

## Abstract

In order to slow the spread of the CoViD-19 pandemic, governments around the world have enacted a wide set of policies limiting the transmission of the disease. Initially, these focused on non-pharmaceutical interventions; more recently, vaccinations and large-scale rapid testing have started to play a major role. The objective of this study is to explain the quantitative effects of these policies on determining the course of the pandemic, allowing for factors like seasonality or virus strains with different transmission profiles. To do so, the study develops an agent-based simulation model, which explicitly takes into account test demand and behavioral changes following positive tests. The model is estimated using data for the second and the third wave of the CoViD-19 pandemic in Germany. The paper finds that during a period where vaccination rates rose from 5 to 40%, seasonality and rapid testing had the largest effect on reducing infection numbers. Frequent large-scale rapid testing should remain part of strategies to contain CoViD-19; it can substitute for many non-pharmaceutical interventions that come at a much larger cost to individuals, society, and the economy.

## Introduction

Since early 2020, the CoViD-19 pandemic has presented an enormous challenge to humanity on many dimensions. The development of highly effective vaccines holds the promise of containment in the medium term. However, most countries find themselves many months—and often years—away from reaching vaccination levels that would end the pandemic or even protect the most vulnerable^1^. In the meantime, it is of utmost importance to employ an effective mix of strategies for containing the virus. The most frequent initial response was a set of non-pharmaceutical interventions (NPIs) to reduce contacts between individuals. While this has allowed some countries to sustain equilibria with very low infection numbers—see Contreras et al.^2^ for a theoretical equilibrium at low case numbers which is sustained with test-trace-and-isolate policies—, most have seen large fluctuations of infection rates over time. Containment measures have become increasingly diverse and now include rapid testing, more nuanced NPIs, and contact tracing. Neither these policies’ effects nor the influence of seasonal patterns or of more infectious virus strains are well understood in quantitative terms.

This paper develops a quantitative model incorporating these factors simultaneously. The framework allows to combine a wide variety of data and mechanisms in a timely fashion, making it useful to predict the effects of various interventions. Behavioral reactions to symptoms or positive tests are explicitly taken into account. We apply the model to Germany, where new infections fell by almost 80% during May 2021. Our analysis shows that, aside from seasonality, frequent and large-scale rapid testing caused the bulk of this decrease, which is in line with prior predictions^3^. We conclude that it should have a large role for at least as long as vaccinations have not been offered to an entire population.

## Model description

**Figure 1:**
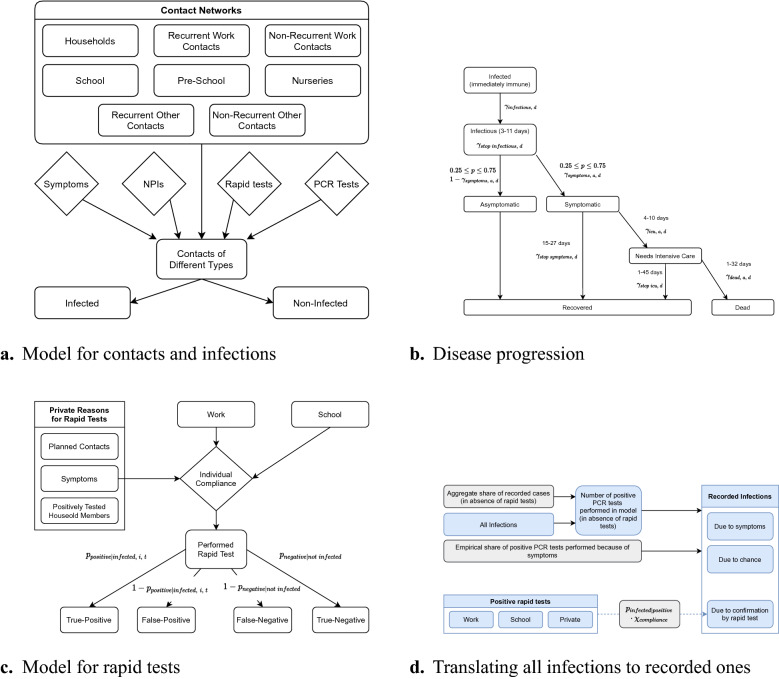
A description of the model can be found in Supplementary Material B. (**a**) The influence of an agent’s contacts to other agents on infections. Demographic characteristics set the baseline number of contacts in different networks ($\eta$). The agents may reduce the number of contacts due to NPIs, showing symptoms, or testing positively for SARS-CoV-2 ($\tau$). Infections may occur when a susceptible agent meets an infectious agent; the probability depends on the type of contact ($\beta _c$), on seasonality ($\kappa _c$), and on NPIs ($\rho _{c,\,t}$). If infected, the infection progresses as depicted in (**b**). If rapid tests are available, agents’ demand is modeled as in (**c**). All reasons trigger a test only for a fraction of individuals depending on an individual compliance parameter; the thresholds for triggering test demand differ across reasons and they may depend on calendar time ($\pi _{c,\,t}$ and $\tau _{c,\,t}$). (**d**) The model of translating all infections in the simulated data to age-specific recorded infections. The model uses data on the aggregate share of recorded cases ($\psi$), the share of positive PCR tests triggered by symptoms ($\chi _{symptom}$), and the false positive rate of rapid tests ($p_{positive|infected,\;i,\,t}$). The lower part of the graph is relevant only for periods where rapid tests are available. All parameters are explained in Supplementary Material A.11.

At the core of our agent-based model^4,5^—we review more literature in Supplementary Material B.1—are physical contacts between heterogeneous agents (Fig. 1a). Each contact between an infectious individual and somebody susceptible to the disease bears the risk of transmitting the virus. Contacts occur in up to four networks: Within the household, at work, at school, or in other settings (leisure activities, grocery shopping, medical appointments, etc.). Some contacts recur regularly, others occur at random. Empirical applications can take the population and household structure from census data and the network-specific frequencies of contacts from diary data measuring contacts before the pandemic^6,7^. Within each network, meeting frequencies depend on age and geographical location (see Supplementary Material A.4).

The four contact networks are chosen so that the most common NPIs can be modeled in great detail. NPIs affect the number of contacts or the risk of transmitting the disease upon having physical contact. The effect of different NPIs will generally vary across contact types. For example, a mandate to work from home will reduce the number of work contacts to zero for a fraction of the working population. Schools and daycare can be closed entirely, operate at reduced capacity—including an alternating schedule—, or implement mitigation measures like masking requirements or air filters^8^. Curfews may reduce the number of contacts in settings outside of work and school. In any setting, measures like masking requirements would reduce the probability of infection associated with a contact^9^.

In the model, susceptibility to contracting the SARS-CoV-2 virus is dependent on age^10,11^. A possible infection progresses as shown in Fig. 1b. We differentiate between an initial period of infection without being infectious or showing symptoms, being infectious (presymptomatic or asymptomatic), showing symptoms, requiring intensive care, and recovery or death (similar to^4^). The probabilities of transitioning between these states depend on age; their duration is random and calibrated to medical literature (for a detailed description see Supplementary Material A.1). Conditional on the type of contact, infectiousness is independent of age^12^.

The model includes several other features, which are crucial to describe the evolution of the pandemic in 2020-2021. New virus strains with different infectiousness profiles may appear. Vaccines may become available. During the vaccine roll-out, priority may depend on age and occupation; vaccine hesitancy is modelled by some individuals refusing vaccination offers. With some probability, vaccinated agents become immune and do not transmit the virus^13–16^.

We include two types of tests. Polymerase chain reaction (PCR) tests reveal whether an individual is infected or not; there is no uncertainty to the result. PCR tests require at least one day to be processed and there are aggregate capacity constraints. In contrast, rapid antigen tests yield immediate results. Specificity and sensitivity of these tests is set according to data analyzed in^17–19^; sensitivity depends on the timing of the test relative to the onset of infectiousness as in^20^. We analyze robustness to different assumptions in Supplementary Material B.12. After a phase-in period, all tests that are demanded will be performed. Figure 1c shows our model for rapid test demand. Schools may require staff and students to be tested regularly. Rapid tests may be offered by employers to on-site workers. Individuals may demand tests for private reasons, which include having plans to meet other people, showing symptoms of CoViD-19, and a household member having tested positively for the virus. We endow each agent with an individual compliance parameter. This parameter determines whether she takes up rapid tests. Positive test results or symptoms lead most individuals to reduce their contacts; this is why tests impact the actual contacts in Fig. 1.

Modelling a population of agents according to actual demographic characteristics means that we can use a wide array of data to identify and calibrate the model’s many parameters (see Supplementary Material A for a complete description). Contact diaries yield pre-pandemic distributions of contacts for different contact types and their assortativity by age group. Mobility data is used to model the evolution of work contacts. School and daycare policies can be incorporated directly from official directives. Administrative records on the number of tests, vaccinations by age and region, and the prevalence of virus strains are generally available. Surveys may ask about test offers, propensities to take them up, and past tests. Other studies’ estimates of the seasonality of infections can be incorporated directly. The remaining parameters—most notably, these include infection probabilities by contact network and the effects of some NPIs, see Supplementary Material A.9—will be chosen numerically so that the model matches features of the data (see^21^ for the general method). In our application, we keep the number of free parameters low in order to avoid overfitting. The data features to be matched include official case numbers for each age group and region, deaths, and the share of the B.1.1.7 strain.

The main issue with official case numbers is that they will contain only a fraction of all infections. In the German case, this specifically amounts to positive PCR tests. We thus model recorded cases as depicted in Fig. 1d. We take mortality-based aggregate estimates of the share of detected cases and use data on the share of PCR tests administered because of CoViD-19 symptoms. As the share of asymptomatic individuals varies by age group, this gives us age-specific shares (see Fig. B.11). Our estimates suggest that—in the absence of rapid testing—the detection rate is 80% higher on average for individuals above age 80 compared to school age children. Once rapid test become available, confirmation of a positive result is another reason leading to positive PCR tests.

## Second and third waves of the CoViD-19 pandemic in Germany

**Figure 2:**
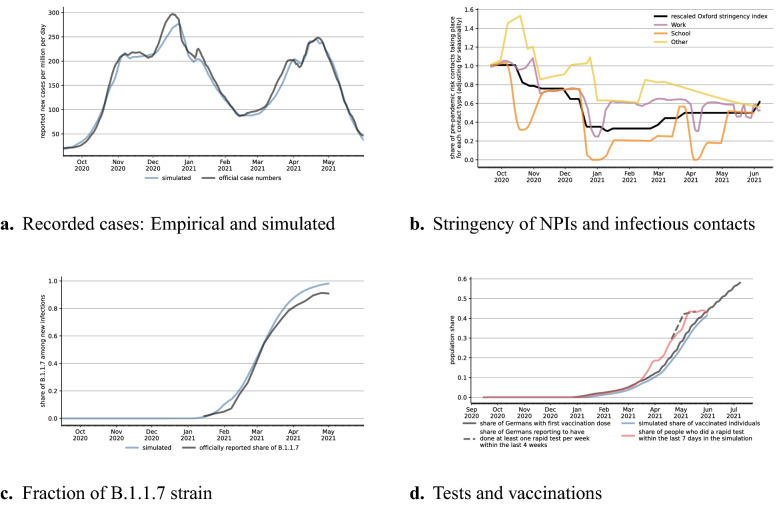
Evolution of the pandemic, its drivers, and model fit, September 2020 to May 2021: Data sources are described in Supplementary Material A. Age- and region-specific analogues to (**a**) can be found in Supplementary Material B.10. For legibility reasons, all lines in (**b**) are rolling 7-day averages. The Oxford Response Stringency Index is scaled as $2 \cdot (1 - x / 100)$, so that a value of one refers to the situation at the start of our sample period and zero means that all NPIs included in the index are turned on. The other lines in (**b**) show the product of the effect of contact reductions, increased hygiene regulations, and seasonality. See Appendix A.5 for separate plots of the three factors by contact type.

The model is applied to the second and third waves of the CoViD-19 pandemic in Germany, covering the period mid-September 2020 to the end of May 2021. Figure 2 describes the evolution of the pandemic and of its drivers. The black line in Fig. 2a shows officially recorded cases; the black line in Fig. 2b the Oxford Response Stringency Index^22^, which tracks the tightness of non-pharmaceutical interventions. The index is shown for illustration of the NPIs, we never use it directly. For legibility reasons, we transform the index so that lower values represent higher levels of restrictions. A value of zero means all measures incorporated in the index are turned on. The value one represents the situation in mid-September, with restrictions on gatherings and public events, masking requirements, but open schools and workplaces. In the seven weeks between mid September and early November, cases increased by a factor of ten. Restrictions were somewhat tightened in mid-October and again in early November. New infections remained constant throughout November before rising again in December, prompting the most stringent lockdown to this date. Schools and daycare centers were closed, so were customer-facing businesses except for grocery and drug stores. From the peak of the second wave just before Christmas until the trough in mid-February, newly detected cases decreased by almost three quarters. The third wave in the spring of 2021 is associated with the B.1.1.7 (Alpha) strain, which became dominant in March (Fig. 2c). Note that we do not model B.1.617.2 (Delta). That variant was first detected in Germany in April; at the end of our simulation period it accounted for less than 5% of cases. In early March, some NPIs were relaxed; e.g., hairdressers and home improvement stores were allowed to open again to the public. There were many changes in details of regulations afterwards, but they did not change the overall stringency index.

By March 2021, the set of policy instruments had become much more diverse. Around the turn of the year, the first people were vaccinated with a focus on older age groups and medical staff (Fig. 2d). Until the end of May, 43% had received at least one dose of a vaccine. In late 2020, rapid tests started to replace regular PCR tests for staff in many medical and nursing facilities. These had to be administered by medical doctors or in pharmacies. At-home tests approved by authorities became available in mid-March. Rapid test centers were opened, and one test per person and week was made available free of charge. In several states, customers were only allowed to enter certain stores with a recent negative rapid test result. These developments are characteristic of many countries: The initial focus on NPIs to slow the spread of the disease has been accompanied by vaccines and a growing acceptance and use of rapid tests. At broadly similar points in time, novel strains of the virus have started to pose additional challenges.

## Results

We draw simulated samples of agents from the population structure in September 2020 and use the model to predict recorded infection rates until the end of May 2021. See Supplementary Materials A.2 and B.9 for details. The blue line in Fig. 2a shows that our model’s predictions are very close to officially recorded cases in the aggregate. This is also true for infections by age and geographical region (see Supplementary Material B.10).

The effects of various mechanisms can be disentangled due to the distinct temporal variation in the drivers of the pandemic. Next to the stringency index, the three lines in Fig. 2b summarize how contact reductions, increased hygiene regulations, and seasonality evolved since early September for each of the three broad contact networks. For example, a value of 0.75 for the work multiplier means that if the environment was the same as in September (levels of infection rates, no rapid tests or vaccinations, only the wildtype virus present), infections at the workplace would be reduced by 25%. Two aspects are particularly interesting. First, all lines broadly follow the stringency index and they would do so even more if we left out seasonality and school vacations (roughly the last two weeks of October, two weeks each around Christmas and Easter, and some days in late May). Second, the most stringent regulations coincide with the period of decreasing infection rates between late December 2020 and mid-February 2021. The subsequent reversal of the trend is associated with the spread of the B.1.1.7 variant. During the steep drop in recorded cases during May 2021, for 42% of the population took at least one rapid tests per week, the first-dose vaccination rate rose from 28% to 43%, and seasonality lowered the relative infectiousness of contacts.

**Figure 3:**
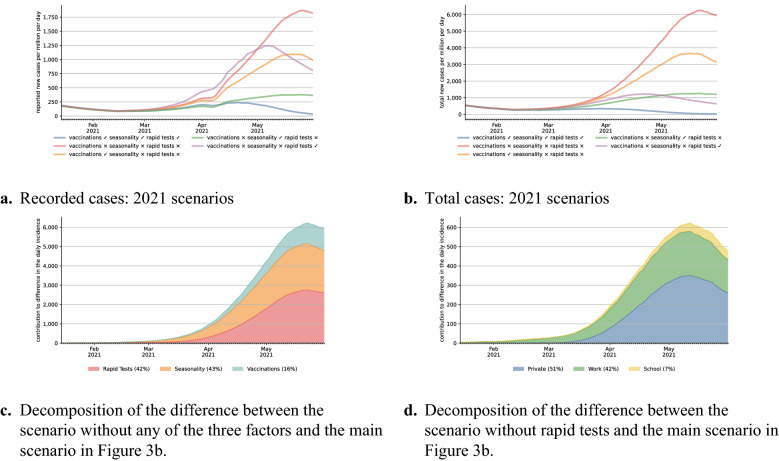
The effect of different interventions on recorded and actual infections. The blue line in (**a**) is the same as in (**a**) and refers to our baseline scenario, so does the blue line in (**b**). The red lines refer to a situation where NPIs evolve as in the baseline scenario and the B.1.1.7 variant is introduced in the same way; vaccinations, rapid tests, and seasonality remain at their January levels. The other scenarios turn each of these three factors on individually. The decompositions in (**c**,**d**) are based on Shapley values, which are explained more thoroughly in Appendix A.10. For legibility reasons, all lines are rolling 7-day averages.

In order to better understand the contributions of rapid tests, vaccinations, and seasonality on the evolution of infections in 2021, Fig. 3 considers various scenarios. NPIs are always held constant at their values in the baseline scenario. Figure 3a shows the model fit (the blue line, same as in Fig. 2a), a scenario without any of the three factors (red line), and three scenarios turning each of these factors on individually. Figure 3b does the same for total infections in the model. Figure 3c employs Shapley values^23^ to decompose the difference in total infections between the scenario without any of the three factors and our main specification.

Until mid-March, there is no visible difference between the different scenarios. Seasonality hardly changes, and only few vaccinations and rapid tests were administered. Even thereafter, the effect of the vaccination campaign is surprisingly small at first sight. Whether considering recorded or total infections with only one channel active, the final level is always the highest in case of the vaccination campaign (orange lines). The Shapley value decomposition shows that vaccinations contribute 16% to the cumulative difference between scenarios. Reasons for the low share are the slow start—it took until March 24th until 10% of the population had received their first vaccination, the 20% mark was reached on April 19th—and the focus on older individuals. These groups contribute less to the spread of the disease than others due to a lower number of contacts. By the end of our study period, when first-dose vaccination rates reached 43% of the population, the numbers of new cases would have started to decline. It is important to note that the initial focus of the campaign was to prevent deaths and severe disease. Indeed, the case fatality rate was considerably lower during the third wave when compared to the second (4.4% between October and February and 1.4% between March and the end of May).

Seasonality has a large effect in slowing the spread of SARS-CoV-2. By May 31, both observed and total cases would be reduced by a factor of four if only seasonality mattered. However, in this period, cases would have kept on rising throughout, just at a much lower pace this is in line with results in^24^, which our seasonality measure is based on. Nevertheless, we estimate seasonality to be a quantitatively important factor determining the evolution of the pandemic, explaining most of the early changes and 43% of the cumulative difference by the end of May.

A similar-sized effect—42% in the decomposition—comes from rapid testing. Here, it is crucial to differentiate between recorded cases and actual cases. Additional testing means that additional infections will be recorded which would otherwise remain undetected. Figure 3a shows that this effect is large and may persist for some time. Until late April, recorded cases are higher in the scenario with rapid testing alone when compared to the setting where none of the three mechanisms are turned on. The effect on total cases, however, is visible immediately in Fig. 3b. Despite the fact that only 10% of the population performed weekly rapid tests in March on average, new infections on April 1 would have been reduced by 53% relative to the scenario without vaccinations, rapid tests, or seasonality. In Supplementary Material B.12, we provide a detailed analysis of whether our results are robust regarding the sensitivity parameters we assume for rapid tests. Even if we take a pessimistic stance, the effect is only reduced from 42% to 38%.

So why is rapid testing so effective? In order to shed more light on this question, Fig. 3d decomposes the difference in the scenario without rapid tests and the main specification into the three channels for rapid tests. Tests at schools have the smallest effect, which is largely explained by schools not operating at full capacity during our period of study and the relatively small number of students (18% of our population are in the education sector, e.g., pupils, teachers; 46% are workers outside the education sector). Almost 40% come from tests at the workplace. Despite the fact that rapid tests for private reasons are phased in only in mid-March, they make up for more than half of the total effect. The reason lies in the fact that a substantial share of these tests is driven by an elevated probability to carry the virus, i.e., showing symptoms of CoViD-19 or following up on a positive test of a household member. The latter is essentially a form of contact tracing, which has been shown to be very effective^2,25,26^. Indeed, a deeper analysis in Supplementary Material B.15 shows that the same amount of rapid tests administered randomly in the population would not have been nearly as effective.

## Discussion and conclusions

**Figure 4:**
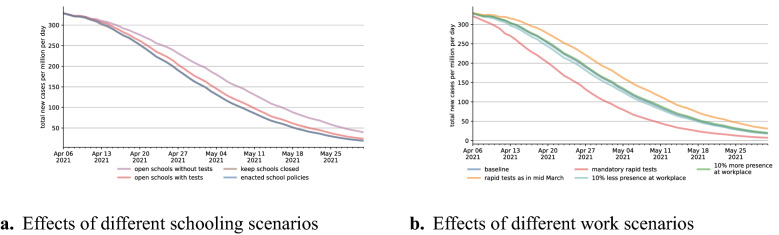
Effects of different scenarios for policies regarding schools and workplaces. Blue lines in both figures refer to our baseline scenario; they are the same as in Fig. 3b. Interventions start at Easter because there were no capacity constraints for rapid tests afterwards. For legibility reasons, all lines are rolling 7-day averages.

Having quantified the effects of various mechanisms, we now simulate hypothetical scenarios comparing changes in NPIs and testing regimes. Two of the most contentious NPIs concern schools and mandates to work from home. In many countries, schools switched to remote instruction during the first wave, so did Germany. After the summer break, they were operating at full capacity with increased hygiene measures, before being closed again from mid-December onward. Some states started opening them gradually in late February, but operation at normal capacity did not resume until the beginning of June. Figure 4a shows the effects of different policies regarding schools starting after Easter, at which point rapid tests had become widely available. We estimate the realized scenario to have essentially the same effect as a situation with closed schools. Under fully opened schools with mandatory tests, total infections would have been 6% higher; this number rises to 20% without tests. These effect sizes are broadly in line with empirical studies (e.g.^27,28^, see Supplementary Material B.11 for a comparison). In light of the large negative effects school closures have on children and parents^29,30^—and in particular on those with low socio-economic status—these results in conjunction with hindsight bias suggest that opening schools combined with a testing strategy would have been beneficial. In other situations, and particular when rapid test are not available at scale, trade-offs may well be different.

Figure 4b shows that with a large fraction of workers receiving tests, testing at the workplace has larger effects than mandating employees to work from home. Whether the share of workers working at the usual workplace is reduced or increased by ten percent changes infection rates by 2.5% or less in either direction. Making testing mandatory twice a week—assuming independent compliance by employers and workers of 95% each—would have reduced infections by 23%. Reducing rapid tests offers by employers to the level of March would have increased infections by 13%.

Our analysis has shown that during the transition to high levels of vaccination and possibly thereafter, large-scale rapid testing can substitute for some NPIs. This comes at a fraction of the cost. A week of the fairly strict lockdown in early 2021 is estimated to have cost around 50 Euros per capita^31^; retail prices for rapid tests were below one Euro in early June 2021 and below five Euros for firms. While we do not distinguish between self-administered rapid tests and point of care rapid tests, the former are likely to play a larger role for indication-driven testing. Widespread availability at low prices seems important. However, they rely on purely voluntary participation in a non-public setting. The benefit of point-of-care rapid tests as a precondition to participate in leisure activities as well as mandatory tests at the workplace or at school come from screening the entire population. This is important because disadvantaged groups are less likely to be reached by testing campaigns relying on voluntary participation (e.g.^32^); at the same time, these groups have a higher risk to contract CoViD-19^33^. Mandatory tests at school and at the workplace will extend more into these groups. The same goes for individuals who exhibit a low level of compliance with CoViD-19-related regulations. Compared to vaccinations, rapid testing programmes allow a much quicker roll-out, making it arguably the most effective tool to contain the pandemic in the short run.

## Supplementary Information

Supplementary Information.

## Data Availability

The source code for the epidemiological model is publicly available in this Github repository: https://github.com/covid-19-impact-lab/sid. The source code of the research project and the data is available in this repository: https://github.com/covid-19-impact-lab/sid-germany.
