# Supplementary material for: The effectiveness of testing, vaccinations and contact restrictions for containing the CoViD-19 pandemic
Source: Sci Rep. 2022 May 16;12:8048. doi: 10.1038/s41598-022-12015-9 (PMC9109202; doi:10.1038/s41598-022-12015-9)
Supplement: Supplementary file 1 — Supplementary Information. [file 41598_2022_12015_MOESM1_ESM.pdf]

Supplementary Material for

The Effectiveness of Testing, Vaccinations and Contact Restrictions for Containing the Covid-19 Pandemic

Janoś Gabler,
Tobias Raabe,
Klara Röhrl,
Hans-Martin von Gaudecker

Contents

Appendix A Materials and Methods **3**
A.1 Course of Disease . . . 3
A.2 The Synthetic Population . . . 5
A.3 Number of Contacts . . . 7
A.4 Assortativity . . . 9
A.5 Policies . . . 10
A.6 Rapid Test Demand . . . 12
A.7 Share of Detected Cases . . . 14
A.8 PCR Testing and Behavioral Response . . . 15
A.9 Estimated Parameters . . . 16
A.10 Shapley Values . . . 21
A.11 Overview Model Parameters . . . 23
A.12 Reproducibility . . . 28

Appendix B Supplementary Text **28**
B.1 Literature Review . . . 28
B.2 Summary . . . 29
B.3 Modeling Numbers of Contacts . . . 31
B.4 Reducing Numbers of Contacts via NPIs . . . 32
B.5 Matching Individuals . . . 33
B.6 Course of Disease . . . 35
B.7 Testing . . . 36
B.8 Seasonality . . . 38
B.9 Initial Conditions . . . 39
B.10 Model Fit . . . 39
B.11 Model Validation . . . 44
B.12 Robustness to assumptions about rapid test sensitivity . . . 47

B.13 Share of Detected Cases 49
B.14 Simulated Rapid Tests 50
B.15 Scenarios 54

Appendix References **57**

Appendix A: Materials and Methods

The model is described by a large number of parameters that govern the number of contacts a person has, the reduction in contacts due to NPIs, the demand for rapid tests and PCR tests, the likelihood of becoming infected on each contact, the likelihood of developing light or strong symptoms or even dying from the disease as well as the duration each stage of the disease takes.

A.1 Course of Disease

This section discusses the parameters governing the course of disease, their sources and how we arrived at the distributions used in the paper. See Figure 1b for a summary of our disease progression model.

The first stage of any disease is the infection. As detailed in Equation B.1 the infection probability depends on the contact type, the calendar date to determine the seasonality, the age group of the susceptible person and the variant the infectious person is carrying. The base infection probability of each contact type (β_c) is estimated inside our model (Section A.9). How we model and calibrate the seasonality effect is detailed in Section B.8. For the susceptibility of each age group (ζ_a) we take the estimates of Davies *et al.* (*1*) (Extended Data Fig. 4). Lastly, we calibrate the infectiousness of the B.1.1.7 variant ($\sigma_{B.1.1.7}$) from Davies *et al.* (*2*) to 1.67.

We denote the latent period—i.e., the time span between infection and the start of infectiousness—by $\gamma_{infectious}$. (*3*) estimate the latent period to last 3.3 days (95% CI: 0.2, 7.9) on average. In line with this estimate our latent period lasts one to five days.

Once individuals become infectious, a share of them goes on to develop symptoms while others remain asymptomatic. We rely on data by (*1*) for the age-dependent probability to develop symptoms. It varies from 25% for children and young adults to nearly 70% for the elderly. Similar to Peak *et al.* (*4*) and in line with He *et al.* (*5*) we set the length of the presymptomatic stage of age group a, $\gamma_{symptoms,a}$ to be one or two days. The probability to become symptomatic for age group a is split equally between one and two days. This combined with our latency period leads to an incubation period that is in line with the meta analysis by McAloon *et al.* (*6*).

We assume that the duration of infectiousness ($\gamma_{stop\ infectious}$) is the same for both symptomatic and asymptomatic individuals as evidence suggests little differences in the transmission rates between symptomatic and asymptomatic patients (Yin and Jin (*7*)) and that the viral load between symptomatic and asymptomatic individuals are similar (Zou *et al.* (*8*), Byrne *et al.* (*9*), Singanayagam *et al.* (*10*)). Our distribution of the duration of infectiousness is based on Byrne *et al.* (*9*). For symptomatic cases they arrive at zero to five days before symptom onset (see their figure 2) and

three to eight days of infectiousness afterwards.[6] Excluding the most extreme combinations, we arrive at 3 to 11 days as the duration of infectiousness.

We use the duration to recovery of mild and moderate cases reported by (*11*, Figure S3, Panel 2) for the duration of symptoms for non-ICU requiring symptomatic cases ($\gamma_{stop\,symptoms}$). We only disaggregate by age how likely individuals are to require intensive care.

For the time from symptom onset until need for intensive care we rely on data by (*12*)) and (*13*) ($\gamma_{icu,\,a}$). For those who will require intensive care we follow Chen *et al.* (*14*) who estimate the time from symptom onset to ICU admission as 8.5 ± 4 days. This aligns well with numbers reported for the time from first symptoms to hospitalization: Gaythorpe *et al.* (*15*) report a mean of 5.76 with a standard deviation of four. We assume that the time between symptom onset and ICU takes four, six, eight or ten days with equal probabilities.

We take the survival probabilities and time to death and time until recovery ($\gamma_{stop\,icu\,a}$ and $\gamma_{dead,\,a}$) from intensive care from Hinch *et al.* (*13*). They report time until death to have a mean of 11.74 days and a standard deviation of 8.79 days. To match this we discretize that 41% of individuals who will die from Covid-19 do so after one day in intensive care, 22% die after twelve days, 29% after 20 days and 7% after 32 days. Again, we rescale this for every age group among those that will not survive. For survivors (*13*) reports a mean duration of 18.8 days until recovery and a standard deviation of 12.21 days. We discretize this such that of those who recover in intensive care, 22% do so after one day, 30% after 15 days, 28% after 25 days and 18% after 45 days.

Individuals can become immune either through infection (γ_{immune}) or vaccination ($\gamma_{vacc,\,d}$). As reinfections are very rare (*16*), we set the immunity period to one year with probability one, i.e. everyone that has been infected enjoys immunity for the rest of the simulation period.

The second route to immunity is through vaccination. Germany has mostly relied on the Pfizer-BioNTech BNT162b2 and Oxford-AstraZeneca ChAdOx1-S vaccines with smaller shares of the Moderna and Johnson&Johnson vaccines (*17*). As Pritchard *et al.* (*18*) and Harris *et al.* (*19*) find no difference in the effectiveness between the two most common vaccines, we do not distinguish between vaccine types.

Immunity is binary in our model, i.e. individuals achieve either sterile immunity or remain susceptible. Thus, we cannot simply use the reported effectiveness but must also include the risk of asymptomatic and sub-clinical reinfection among the vaccinated in our probability to become immune upon vaccination. This is important as there is ample evidence by now that vaccinated individuals can still get infected with SARS-CoV-2 and transmit the disease (*19*–*21*).

6. Viral loads may be detected much later but eight days seems to be the time after which most people are culture negative, as also reported by Singanayagam *et al.* (*10*).

The reported effectiveness for BNT162b2 is estimated to be 90% 21 days after the first shot (*22*). The effectiveness does not increase much through the booster shot as Thompson *et al.* (*23*) report 90% (95% CI = 68%–97%) effectiveness against PCR-confirmed infections after two doses for mRNA vaccines in general. We therefore do not distinguish between the first and the booster shot.

On the other hand, Lipsitch and Kahn (*24*) report a lower bound on transmission for the very similar Moderna vaccine of 61%.

To strike a middle ground we assume that 75% of individuals achieve sterile immunity after vaccination. This is split into 35% reaching immunity after 14 days after the first shot and 40% reaching immunity after 21 days.

A.2 The Synthetic Population

We build a synthetic population based on the German microcensus (*25*). We only use private households, i.e. exclude living arrangements such as nursing homes as non-private households vary widely in size and it is very difficult to know which contacts take place in such living arrangements.

We sample households to build our synthetic population of over one million households keeping for each of the 2.3 million individuals their age, gender, occupation and whether they work on Saturdays and Sundays. For each household we draw its county and set the corresponding federal state.

We randomly assign 35% of children below three to attend a nursery (*26*). For children between three and six years old, we assume all go to preschool (officially 92.5% according to (*26*)). Children that attend a nursery meet in groups of four (*27*) plus one adult care taker every weekday when there are no school vacations. Preschool children meet in groups of nine (*27*) with two adult care takers. These groups are mixed with respect to age but all belong to the same state and mostly to the same county.

Every child that goes to school is part of a school class. Each school class meets three times per weekday, each time with a different set of two teachers, unless there are vacations or policies that suspend schools.[7] Each class consists of approximately 23 students (*28*). All students in a class are of the same age and live in the same state and mostly also in the same county. In addition, each child gets assigned a value that captures his or her need to attend nursery, preschool or school. This allows us to capture various degrees of emergency care that can be granted while educational facilities are closed or are on some kind of rotating schedule.

Workers are assigned to a daily meeting work group. The group sizes vary to match the number of daily repeating work contacts reported by working individuals in (*29*). These groups only consist of workers that work in the same county. For

7. We implement vacations on the federal state level.

a distribution of the number of daily recurring work contacts see Figure A.2e. To match the number of weekly work groups we match each worker with up to 14 other workers into pairs to match the number of reported weekly work contacts shown in Figure A.2f. Each pair is assigned a weekday on which they always meet in the absence of work policies. 80% of these contacts are individuals from the same county. In the same way children have an educational priority determining if they are entitled to emergency care workers are assigned a work contact priority that captures how necessary their work is and to which degree they can work from home. This means that it's always the same individuals that continue to have work contacts when work from home mandates of a certain strictness are in place.

In addition to creating groups for educational facilities and work we also have other recurring contacts to represent things like groups of friends or sports teams that practice regularly together. Both daily and weekly groups are created analogously to the work groups but matching the numbers in Figure A.2b and Figure A.2c. In addition, since leisure contacts are highly assortative by age all individuals that have a daily leisure contact are matched with a person not only from the same county but also from the same age group.

The individuals in our population can react to events such as developing symptoms that are typical of CoViD-19, a positive PCR test or a positive rapid test by reducing their contacts. To determine who would reduce their contacts in such a situation or demand a rapid test we introduce a quarantine compliance parameter. Similarly, we introduce a rapid test compliance parameter that determines in which order individuals start demanding rapid tests when rapid tests become increasingly available. This makes sure that when for example only 10% of workers get tested, it's the same workers that have access to tests every week.

Lastly, for the distribution of vaccinations every individual is assigned a vaccination group and a vaccination rank from that group that creates a complete vaccination queue over the population including a share that refuses to be vaccinated (ξ) which we calibrate to 15% (*30*). The vaccination groups are created to match the recommendations by the Ständige Impfkommission (*31*).[8] To cover that the Pfizer-BioNTech vaccine was later approved for younger age groups we put adolescents and children into two groups that follow after the general population. These groups do not become eligible within our simulation frame until June. The way vaccinations are rolled out in our model is shown in Figure A.1.

8. We cover that teachers were prioritized more than recommended by the commission.

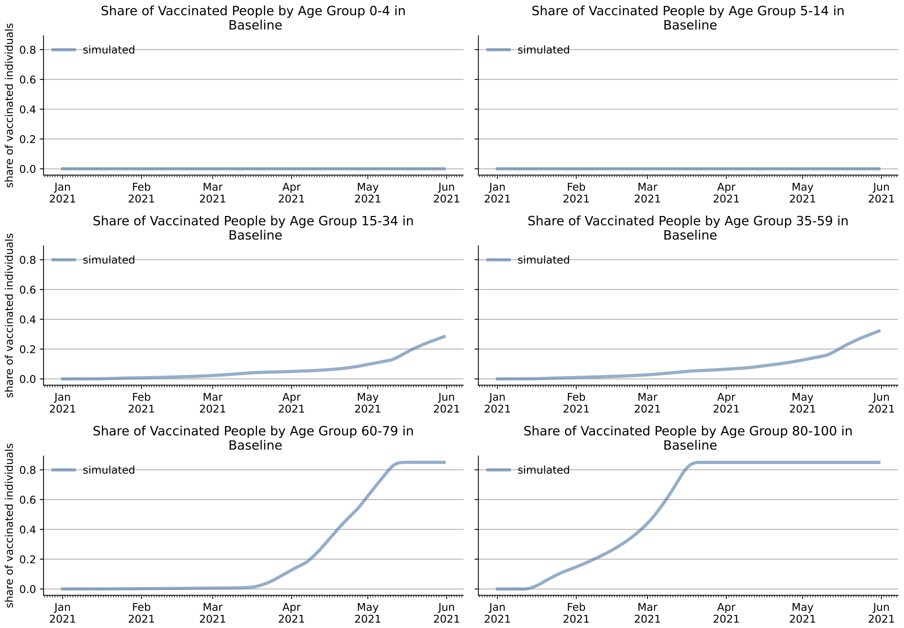

Figure A.1. Vaccination Rates by Age Group

Note: An individual's vaccination priority depends on her work contact priority, her age group and a random component to capture preconditions like diabetes. 15% of the population refuse to be vaccinated (ξ). Adolescents would be vaccinated after the general population and children last. The figure clearly shows that the first vaccinations go to some workers with very high work contact priority and to the 80 to 100 age group followed by the 60 to 79 year olds. Both groups are saturated with vaccinations by mid March and start of May respectively. By June a third of the younger adults have received the vaccination but these groups still remain far from herd immunity thresholds.

A.3 Number of Contacts

We calibrate the parameters for the predicted numbers of contacts from contact diaries of over 2000 individuals from Germany, Belgium, the Netherlands and Luxembourg (*29*). Each contact diary contains all contacts an individual had throughout one day, including information on the other person (such as age and gender) and information on the contact. Importantly, for each contact individuals entered of which type the contact (school, leisure, work etc.) was and how frequent the contact with the other person is. Binning the number of contacts for very high numbers, we arrive at the distributions of the numbers of contacts by type of contact (η_c) as shown in Figure A.2.

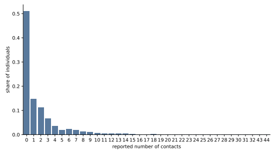

(a) Number of Non Recurrent Other Contacts

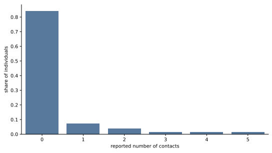

(b) Number of Daily Recurrent Other Contacts

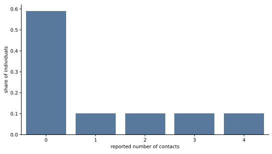

(c) Number of Weekly Recurrent Other Contacts

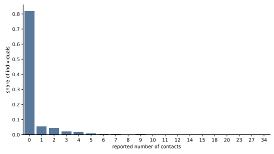

(d) Number of Non Recurrent Work Contacts

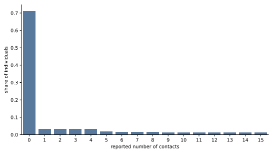

(e) Number of Daily Recurrent Work Contacts

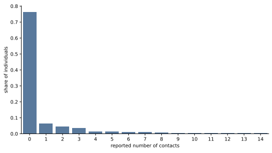

(f) Number of Weekly Recurrent Work Contacts

Figure A.2. Number of Contacts of the Different Contact Types

Note: This figure shows the pre-pandemic number of contacts individuals report of different contact types (η_c). In the model it is sampled every day which of the numbers of non recurrent contacts a person is planned to have. Note that the contact diaries include such high values that super spreading events are well possible in our model through non recurrent models. For recurrent contacts individuals are put into groups that meet either every day or on a particular week day every day. The upper row shows the distribution of the number of other contacts individuals report (η_{other}). Other contacts include all contacts that are not household members, school contacts or work contacts, for example leisure contacts. We assume that individuals in households with children or teachers or retired individuals have additional non recurrent other contacts during school vacations to cover things like family visits or travel during vacations. The lower row shows the distribution of the different types of work contacts (η_{work}). Work contacts only take place between working individuals.

An exception where we do not rely on the data by (*29*) are the household contacts. Since households are included in the the German microcensus (*25*) on which we build our synthetic population we simply assume for the household contacts that individuals meet all other household members every day. The number of household contacts that happen every day is shown in Figure A.3.

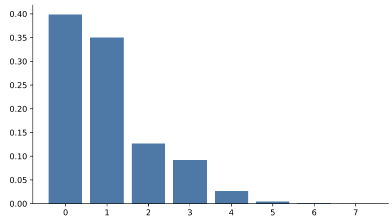

Figure A.3. Number of Household Contacts

Note: Every individual meets all other household members every day. The German microcensus sampled full households such that our synthetic population automatically fits population characteristics such as size and age distribution.

A.4 Assortativity

As explained in section B.5, the probability that two individuals are matched can depend on background characteristics. In particular, we allow this probability to depend on age and county of residence (α). While we do not have good data on geographical assortativity and set it such that 80% of contacts are within the same county, we can calibrate the assortativity by age from (*29*).

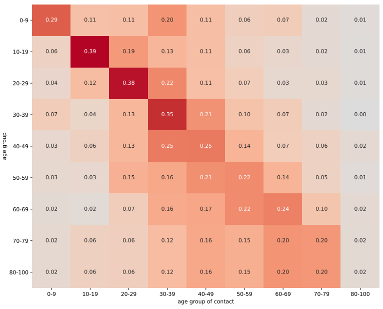

(a) Distribution of Non Recurrent Other Contacts by Age Group

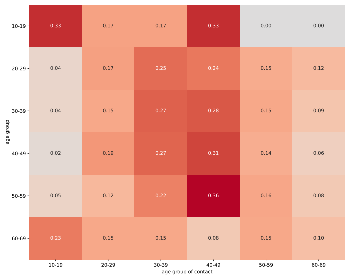

(b) Distribution of Non Recurrent Work Contacts by Age Group

Figure A.4. Assortativity by Age Group for Non Recurrent Other and Work Contacts

Note: The figure shows the distribution of non recurrent contacts by age group for other contacts on the left and work contacts on the right. A row shows the share of contacts a certain age group has with all other age groups. Higher values are colored in darker red tones. The diagonal represents the share of contacts with individuals from the same age group. The 80-100 age group for other contacts was so small that we assumed for them to have the same contact distribution as the 70-79 year olds. For work contacts, we only show age groups that have a significant fraction of working individuals.

Figure A.4a shows that assortativity of the other contacts by age is especially strong for children and adolescents. For older people, the pattern becomes more dispersed around their own age group, but within-age-group contacts are still the most common contacts. Figure A.4b shows that assortativity by age is also important among work contacts.

For recurrent contacts, we constructed groups to have the following features: Recurrent work contacts are not assortative by age. Daily work groups are always of the same county and weekly work contacts are to 80% with workers from the same county. Other recurrent contacts are constructed the same way but we impose for daily contacts that they are always with individuals from the same age group. School classes are groups where the same children of the mostly same age and county meet with teachers every day. Nurseries and preschools mix children by age but match them to come mostly from the same county. Household age composition follows directly from the German microcensus data we use to construct our synthetic population.

A.5 Policies

Our policies (denoted by ρ) usually affect one of three contact types: education, work and other contacts. Germany had no policies limiting contacts within households so there are no policies on them in our model.[9]

For nurseries, preschools and schools we implement vacations as announced by the German federal states as well as school closures, emergency care and rotating schedules where only one half of students attends every other week or day. An approximation of the share of contacts still taking place with the different school regulations can be found in Figure A.5a. Note that schooling policies differ between states and usually involve rules that depend on local incidences. We simplify these rules to one federal policy from the federal incidence and the policies of the three most populous federal states (North Rhine-Westphalia, Bavaria and Baden-Württemberg). The testing policies for schools are described in Section A.6.

Until November schools were open normally. Starting in November, we assume that increased hygiene measures were taken. Schools stayed open until mid December. From mid December until January 10 schools closed and only offered so called "emergency care" for young children whose parents could credibly demonstrate that both had to work and had no other child care arrangement. Approximately 25% of primary school children and 5% of secondary students attended school as a result. After January 10 when parents had returned to work the rules for emergency care were relaxed and approximately a third of primary school children and 10% of secondary students attended school as a result. In addition, graduating classes (most adolescents between 16 and 18) were allowed to return to school in a rotating scheme where each class was split in two groups. Relying on anecdotal evidence we assume that the groups rotate on a daily basis.Starting on February 22 primary school children were also allowed to return to school on a rotating basis until mid March. We summarize the school policy from mid March until Easter as all students being on a rotating school schedule. In addition, children that qualify for emergency care also attend on days where their group is scheduled to not attend school physically. After the Easter break schools were mostly closed again. Part of this was a federal law, the so called "Bundesnotbremse" (*32*) that set rules for schools based on local incidences that were binding at the time. As a result, most states adjusted their schooling policies and during April most schools were closed with emergency care arrangements as in the time from January 10 to February 21. As cases fell schools were allowed to gradually open. We summarize this as students being on the same rotating schedule as from mid March to Easter starting on May 1 (*33*–*41*).

The policies for preschools and nurseries are similar to the school policies but simpler. Until November children attended completely normally, starting in Novem-

9. Household contacts can, however, be reduced when individuals quarantine themselves after developing symptoms, for example. This happens to a lesser degree than other contacts to capture difficulties in isolation within the home.

ber with increased hygiene measures. Nurseries and preschools stayed open until mid December. From mid December until January 10, nurseries and preschools were nearly completely closed. If parents could credibly demonstrate that both parents work in systemically relevant professions and no other child care arrangement was possible, nurseries and preschools offered so called “emergency care”. We assume 10% of children qualified and used emergency care during this time. After January 10 when parents had returned to work the rules for emergency care were relaxed and we assume a third of children attended nursery and preschool. This policy stayed in place until February 20. Afterwards, preschools and nurseries were open normally (maintaining increased hygiene measures) until mid March. Then during the third wave the restrictions February were put back into place until end of April when nurseries and preschools opened again and stayed open for the rest of our simulation period - maintaining increased hygiene measures. (*35*, *42*–*46*).

For work contacts we use the reductions in work mobility reported by the Google Mobility Data (*47*) to calibrate the reduction in physical work contacts ($\rho_{w,\,attend,\,t}$). Reductions in work contacts are not random but governed through a work contact priority where the policy changes the threshold below which workers stay home. Figure A.5b shows the share of workers that go to work over time at the federal German level. We use the data on the state level to account for local holidays and differences in state regulations. In addition, for both work and school contacts we assume that hygiene measures (such as masks, ventilation and hand washing) became more strict and more conscientiously observed in November 2020, leading to a reduction of 33% in the number of contacts with the potential to transmit Covid-19 ($\rho_{hygiene}$).

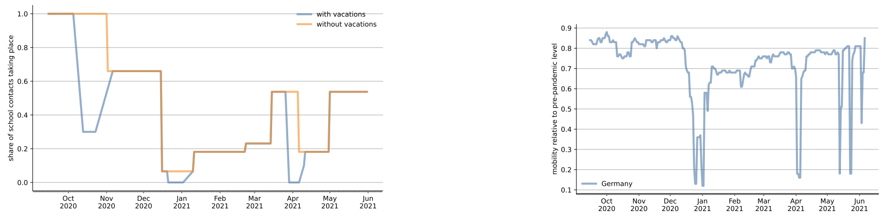

(a) School Attendance

(b) Work Attendance

Figure A.5. The Contact Reduction Effects of School and Work Attendance Policies

Note: The left figure shows the approximate share of school contacts taking place with and without vacations factored in. In contrast to other policies, school policies are not implemented via multipliers but as mechanistic models (e.g. split class approaches in with emergency care). For the above plot we assigned approximate multipliers to those policies. The figure is, thus, only an illustration that shows the approximate share of contacts taking place compared to the pre-pandemic level with and without vacations. The right figure shows the work mobility as reported by (*47*). We take this as a proxy of the share of workers who still have physical work contacts ($\rho_{w,\,attend,\,t}$). The figure interpolates over weekends as we handle weekend effects through information on work on weekends in the German census data we use. The figure shows the share for Germany as a whole. To capture the effect that local policies, school vacations, etc. have on work contacts we use the data on the state level to determine which workers go to work depending on the state they live in.

Lastly, for the other contacts category ($\rho_{other,t}$) we could not calibrate the policies from data but estimated the policy effects. The estimation and values are detailed in Section A.9 and Figure A.10.

A.6 Rapid Test Demand

In our model, there are five reasons why rapid tests are done:

(1) someone plans to have work contacts

(2) someone is an employee or student of an educational facility

(3) a household member has tested positive or developed symptoms

(4) someone has developed symptoms but has not received a PCR test

(5) someone plans to participate in a weekly non-work meeting

For work contacts, we know from the COSMO study ((*48*), 20th/21st of April) that 60% of workers who receive a test offer by their employer regularly use it ($\pi_{w,d}$). We assume this share to be time constant.

In addition, there are some surveys that allow us to trace the expansion of employers who offer tests to their employees ($\pi_{w,s,t}$). Mid march, 20% of employers offered tests to their employees (*49*). In the second half of March, 23% of employees reported being offered weekly rapid tests by their employer (*50*). This share increased to 61% until the first days of April (*51*, *52*). Until mid April 72% of workers were expected to receive a weekly test offer (*51*, *52*). However, according to surveys conducted in mid April (*48*), less than two thirds of individuals with work contacts receive a test offer. Starting on April 19th employers were required by law to provide two weekly tests to their employees (*53*). We assume that compliance is incomplete and only 80% of employers actually offer tests. We interpolate between these points linearly, arriving at the blue line in Figure A.6. In addition, we increase the frequency of testing ($\theta_{t,work}$) from weekly to twice weekly during April.

We assume that employees in educational facilities start getting tested in 2021 and that by March 1st 30% of them ($\pi_{teacher,t}$) are tested weekly ($\theta_{before\,Easter,educ} = 7$). The share increases to 90% for the week before Easter. At that time both Bavaria (*54*) and Baden-Württemberg (*55*) were offering tests to teachers and North-Rhine Westphalia (*56*) and Lower Saxony (*57*) were already testing students and tests for students and teachers were already mandatory in Saxony (*58*). After Easter we assume that 95% of teachers get tested twice per week ($\theta_{after\,Easter,educ} = 3$).

Tests for students started later (*55*, *56*) so we assume that they only start in February and only 10% of students get tested by March 1st ($\pi_{students,t}$). Relying on the same sources as above we approximate that by the week before Easter this share had increased to 40% (*56*). After Easter the share of students receiving twice weekly tests is set to 75%. This is based on tests becoming mandatory in Bavaria (*59*) and North Rhine-Westphalia (*60*) after their Easter breaks and on the 19th

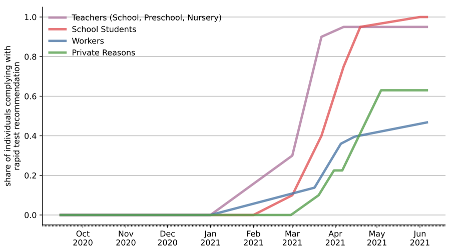

Figure A.6. Share of Individuals Doing a Rapid Test.

Note: Rapid test demand can be triggered by individuals planning to have education contacts ($\pi_{teacher,\,t}$ or $\pi_{students,\,t}$), work contacts ($\pi_{w,\,d}$ and $\pi_{w,\,s,\,t}$), developing symptoms without access to a PCR test, having a household member with a positive test or symptoms ($\pi_{private,\,t}$). In each case whether a rapid test is done depends on how long it has been since the individual's last rapid test and her individual compliance parameter. As an example, take a worker in May. In that time workers are encouraged to test themselves twice weekly (or every three days, i.e. $\theta_{May,\,work} = 3$) but there is no general requirement to test themselves. If the worker has not done a test within the last four days in our model she will demand a test if her (time-constant) compliance parameter belongs to the upper 60% in the population.

in Baden-Württemberg (*61*), after which we assume twice weekly rapid tests to be mandatory for all students in Germany. Again, we interpolate linearly between these points and arrive at the purple line for teachers and the red line for school students in Figure A.6.

To limit our degrees of freedom, we only have one parameter that governs how many individuals do a rapid test because of any of the private demand reasons ($\pi_{private,\,t}$).[10] We assume that there is no private rapid test demand until March when both the citizens' tests and rapid tests for lay people started to become available (*62*, *63*) and other access to rapid tests was very limited.

According to the COSMO study (*64*) 63% would have been willing to take a test in the round of 23rd of February 2021 when an acquaintance would have tested positive. Since this is only asking for willingness not actual behavior, we take this as the upper bound of private rapid test demand which we estimate in our model to be reached in the beginning of May. To cover that many people are likely to have sought and done their first rapid test before the Easter holidays we add another point that we estimate for the rapid test demand around Easter. Similarly, we estimate one point in mid March when tests started to become available in grocery stores and pharmacies which we estimate in our model. The resulting share of private rapid test demand is shown as the green line in Figure A.6 (also see Section A.9 for details on the estimation).

10. The reasons that can lead to an individual doing a rapid test for private reasons are own symptoms but no PCR test, planned weekly leisure meeting or a symptomatic or positively tested household member

A.7 Share of Detected Cases

One important feature of our model is that we distinguish between undetected and detected cases and that we model which cases are detected and which are not (see Section B.7 for a detailed description for how we model both rapid and PCR tests). For our model it is important to have an estimate for the share of cases that is detected in the absence of rapid tests (ψ_t). For this we rely on the (Dunkelzifferradar Project *65*) which uses estimates of the case fatality rate to estimate the number of total cases given the number of CoViD-19 deaths which are assumed to be perfectly observable. For 2020, we follow the reported share of detected cases quite closely. One exception is the phase of November 2020 where we interpolate to maintain monotonicity during the fall as there was no reason why the share of detected cases should have risen in that time[11]

Since vaccinations started after Christmas 2020 and these were predominantly given to nursing homes in the beginning and other vulnerable groups in spring, we expect the relationship between deaths and the number of total infections to change rapidly in 2021. This is why we stop using the share of detected cases estimated by the Dunkelzifferradar after Christmas. Instead, we assume that the share of detected cases would have stayed the same in the absence of rapid tests. Thus, we also achieve in our model an increase in the share of detected cases but this is driven from inside our model through increased rapid testing which lead follow-up PCR tests when they are positive (see Section B.13 and B.7).

Lastly, we model reductions in the share of detected cases due to the two major holidays in our simulation period, Christmas and Easter. During both holidays many laboratories did not process tests and most physicians' offices were closed, leading to less PCR tests and short and large drops in the share of known cases. The resulting share of detected cases in the absence of rapid tests is shown in Figure A.7 and was estimated to fit the data.

11. The testing policy changed in November (*66*). However, this only moved the rare PCR tests more towards vulnerable groups.

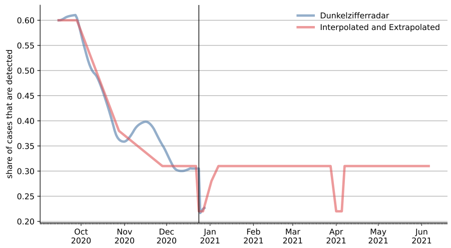

.

Figure A.7. Share of Detected Cases in the Absence of Rapid Tests

Note: The figure shows the share of cases that is reported as an official case via PCR confirmation. We use the overall share of known cases that was estimated through the case fatality ratio by the (Dunkelzifferradar Project *65*) for all of 2020 and then assume it to be constant as vaccinations of the elderly strongly affect the case fatality rate which the project does not account for. Starting in 2021 in addition to the overall numbers of detected cases through symptoms and a random component, cases are also detected through confirmation of positive rapid tests which happens endogenously inside the model. For the public holidays of Christmas and Easter we lower the share of detected cases as fewer PCR tests are available during public holidays. See Figure B.11 for how the share of detected cases develops in our model for each age group

A.8 PCR Testing and Behavioral Response

This section describes the remaining parameters for our testing model. Refer to Section B.7 for a description of the full testing model.

From the share of detected cases and the number of infections we arrive at the number of positive PCR tests in our model. A share of these positive tests goes to symptomatic individuals ($\chi_{symptom,\,t}$). This share is calibrated from German data on case characteristics (*67*) and shown in Figure A.8. We keep $\chi_{symptom,\,t}$ constant after Christmas because the RKI data does not include if a PCR test was done to confirm a positive rapid test and this share is used for PCR test demand without prior rapid test indication.

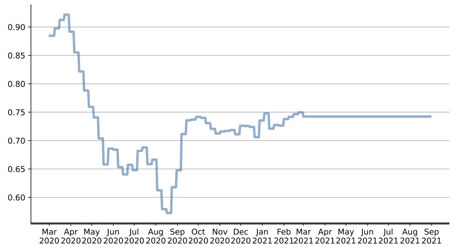

Figure A.8. Share of Positive PCR Tests Administered to Symptomatic Individuals

Note: The share of positive PCR tests that are administered to symptomatic individuals ($\chi_{symptom,\,t}$). Since it was not recorded for every case if the person was symptomatic or not we take the midpoint between the upper and lower bound. We keep the share constant after Christmas because the RKI data does not include if a PCR test was done to confirm a positive rapid test and this share is used for PCR test demand without prior rapid test indication.

PCR tests take one to four days until their result is revealed to the individual ($\gamma_{PCR,d}$). Relying on the ARS data (*68*) we calculate that 33% of individuals receive the test result after one day, 50% after two days, 10% after three days and 7% after four days.

To model the demand for PCR tests through rapid tests, we only need the share of individuals that seek a PCR test to confirm a positive rapid test result ($\chi_{confirmation}$). We calibrate this from Betsch *et al.* (*48*) who asked this as a hypothetical question in March of 2021. There 82% of Germans reported that they would follow up on a positive rapid test with a PCR test.

Lastly, we need to set the parameters that decide how individuals reduce their contacts after certain events, τ. We distinguish between the reduction in household contacts (which are harder to avoid) and non household contacts. There are three events which trigger potential contact reductions: showing symptoms of CoViD-19, having received a positive rapid test and having received a positive PCR test. The only survey data we are aware of on this is Betsch *et al.* (*48*) where 85% of individuals claimed they would isolate and restrict their contatcs after a positive rapid test. We assume this reduction for non household contacts. As household contacts are much more difficult to avoid, we assume that they are only reduced by 30%. We assume the same behavior for individuals that develop symptoms. Lastly, we assume the response to a positive PCR test to be stronger than in the other two cases and set the reduction of non household contacts to 95% and the reduction of household contacts to 50%.

A.9 Estimated Parameters

We estimate parameters that cannot be calibrated outside of the model with the method of simulated moments (*69*) by minimizing the distance between simulated and observed infection rates (disaggregated by region and age groups) and fatality rates. Since our model includes a lot of randomness, we average simulated infection rates over several model runs.

All estimated parameters are described in Table A.1.

Table A.1. Estimated Parameters

notation	estimate	note
**Infection Probabilities**		
$\beta_{household}$	0.1	base probability of getting infected by an infectious household member
β_{school}	0.012	base probability of getting infected by an infectious classmate or teacher
$\beta_{young\ educ}$	0.005	base probability of getting infected by an infectious classmate or teacher
β_{work}	0.1475	base infection probability for work contacts
β_{other}	0.15875	base infection probability for other contacts
**Policy Parameters**		
$\rho_{hygiene}$	0.66	reduces infectiousness of work and education contacts from November to end of simulation
$\rho_{other,\ before\ Oct\ 1}$	0.75	before October
$\rho_{other,\ Oct\ 1\ to\ Oct\ 20}$	1.00	high activity due to reopenings and fall vacations
$\rho_{other,\ Oct\ 21\ to\ Nov\ 1}$	0.75	anticipation of a lockdown and precaution due to high incidenes
$\rho_{other,\ Nov\ 2\ to\ Dec\ 1}$	0.52	"lockdown light"
$\rho_{other,\ Dec\ 2\ to\ Dec\ 23}$	0.57	"lockdown light" with lockdown fatigue and holiday shopping
$\rho_{other,\ Dec\ 24\ to\ Dec\ 26}$	0.65	Christmas holidays
$\rho_{other,\ Dec\ 27\ to\ Feb\ 10}$	0.35	hard lockdown after Christmas
$\rho_{other,\ Feb\ 11\ to\ Feb\ 28}$	0.50	lower precaution due to low incidences and lockdown fatigue
$\rho_{other,\ after\ Feb\ 28}$	0.515	many contact reducing policies are lifted
**B.1.1.7 Introduction**		
$\omega_{B.1.1.7,\ Jan\ 31}$	0.986	number of B.1.1.7 cases per 100 000 individuals to import on January 31st. Imported B.1.1.7 cases gradually rise from January 1st where 0 cases are imported. No cases are imported in other months.
**Rapid Test Introduction**		
$\pi_{private,t}$	Figure A.6	the private rapid tests levels in mid March and at Easter as well as the date at which full availability of private rapid tests is reached are fit to the data. Values between those levels are interpolated linearly. The remaining rapid tests demands are calibrated from surveys. See Section A.6

We fit our model to data for Germany from mid September 2020 until June 2021. We do not use earlier periods for three reasons. Firstly, in the beginning PCR tests were very scarce and the reported case numbers unreliable. Secondly, during the summer the case numbers were extremely low. This could lead to the epidemic going extinct in our simulation. Thirdly, over the summer, imported cases from touristic travel were likely important for the infection dynamic but there is not enough data to include them into our model.

To avoid over-fitting and simplify the numerical optimization problem, we only allow for five different infection probabilities: 1) for contacts in schools 2) for contacts in preschools and nurseries. 3) for work contacts. 4) for households. 5) for other contacts.

Since the infectiousness of a contact between an infectious and a susceptible person depends on many things, the numerical values of the infection probabilities in Table A.1 only reflect a base probability. This base probability is modified by a seasonality factor, an age specific susceptiblity factor and an infectiousness factor that depends on the virus strand of the infected person. The base infection probability is only equal to the actual infection probability when all of those factors are 1. This would be the case for a contact between an 80+ year old susceptible person with a person who is infected with the B.1.1.7 strand of the virus on January first.

It is not possible to rank different types of contacts according to their infectiousness just from the numerical values of the infection probabilities. There are two reasons for this: Firstly, for computational reasons the seasonality factor is normalized such that it reaches 1 at its peak. It has thus a lower average for contact types with strong seasonality (e.g. other contacts) than for contact types with weak seasonality (e.g. work contacts). Secondly, for household and school contacts we do not have data on whether people actually have physical contact. Thus the infection probabilities for those contact types are actually the product of the probability to actually have physical contact on a given day and the infection probability of that contact.

In order to get a feeling for the infectiousness of each contact type it is more intuitive to look at how many infections were actually caused by each contact type. This is depicted in Figure A.9. We can see that work and other contacts are the main drivers of the pandemic, followed by infections in households. Schools and preschools contribute fewer infections which is to be expected given that there are much fewer students than working adults in the German population. Nevertheless, Figure B.16 shows that schools do have a notable effect on the infection dynamic in the long run.

We also estimate a parameter that reflects the effect of hygiene measures at work and in educational facilities. This parameter becomes active in November 2020 when stricter mask mandates and distancing rules were introduced. It is estimated to reduce infectiousness of contacts by one third.

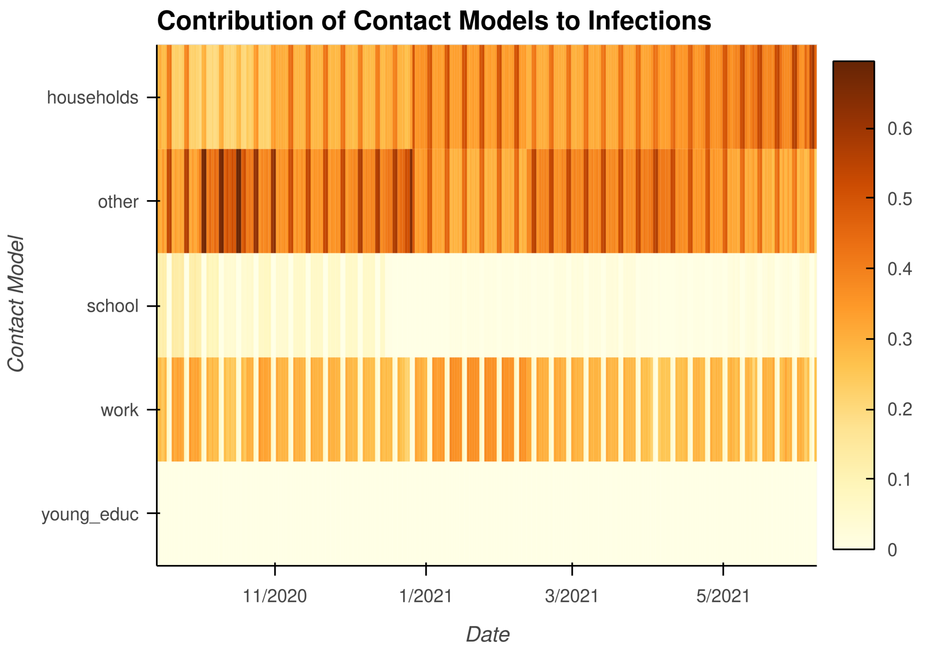

Figure A.9. Daily share of infections by contact type

Note: Daily share of infections that were contributed by each contact type. Darker colors mean that a larger share of infections were contributed by that contact type. The majority of infections take place in the workplace, in households and via other contacts. Schools and preschools contribute less infections, especially after hygiene measures have been introduced.

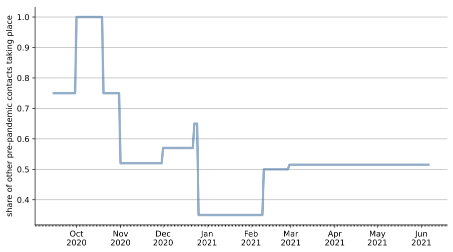

Figure A.10. Share of Pre-Pandemic Other Contacts Taking Place with Infection Potential

Note: Values of the other multiplier. All values are estimated via the method of simulated moments. The rationale behind each switching point is described in Table A.1

Moreover, we estimate nine different multipliers that reflect how strongly other contacts are reduced over time. The dates at which we switch between the multipliers usually coincide with policy changes and is not determined from the case numbers. The only exception to this are slight adjustments to parameters to incorporate lockdown fatigue (towards the end of a lockdown period) or precautionary contact reductions (in times of high incidences right before a lockdown is enacted). The estimated other multipliers are also depicted in Figure A.10.

While we estimate nine different values for the other contact multiplier, they are not estimated completely freely. In particular we ensure that the ordering of the parameter values is consistent with the stringency of policies. For example, the strongest contact reduction was estimated for January 2021 during where very strict measures and curfews were in place, whereas the weakest contact reduction was in October 2020 where policies were very lenient.

Since we do not have good data on the reduction of other contacts, it is not possible to separately estimate parameters for contact reduction and the effect of hygiene measures. The reported other multipliers in Table A.1 are thus a combination of contact reduction and hygiene measures.

Finally we estimate one parameter that governs the introduction of the B.1.1.7 virus variant in January 2021. This parameter implies that at the end of January roughly one case per 100 000 individuals per day is imported. After January we do not model imported cases of B.1.1.7 anymore because they are negligible compared to the endogenous growth of that virus variant.

While a formal identification argument is beyond the scope of this paper, below we give a rough intuition which features of the data help us to estimate each parameter.

The different infection probabilities can be separately identified because the degree to which each contact type is active varies over time (e.g. school closures, vacations and different work from home policies) and they affect different subgroups of the population differently (e.g. β_{school} most strongly affects kids whereas β_{work} has the strongest effect on adults in working age and β_{other} affects all age groups

equally). The hygiene and other multipliers can be identified because they are only active in certain time periods. However, it is necessary to normalize one other multiplier to 1 because there is no period without any contact reduction in our data. The introduction parameter for the B.1.1.7 mutation can be identified from the share of that virus strand in the population. The rapid test demand parameters are identified because rapid tests first lead to a very steep increase in observed cases and then to a sudden decrease – in a time where almost all other things in the model would not cause a change in trend.

A.10 Shapley Values

We decompose the effects of different NPIs and seasonality on the infection rates with Shapley values. Shapley values (*70*) are a concept in game theory to divide payoffs between a coalition of players. It allows to assign a single value to the contribution of an NPI or seasonality which takes into account substitutional and complementary effects with other factors.

More formally, define a coalitional game with N players and a super-additive function v which maps subsets of N to the real numbers. The function v is also called the characteristic function and assigns a value to a coalition. Then, the Shapley value ϕ for player i is

$$\phi_i(v) = \frac{1}{|N|!} \sum_{S \subseteq N \setminus \{i\}} |S|!(|N| - |S| - 1)!(v(S \cup \{i\}) - v(S))$$

The last term $(v(S \cup \{i\}) - v(S))$ is the marginal contribution of player i minus the coalition without player i. Then, compute the sum of marginal contributions over all subsets S of N which do not include player i. Each marginal contribution has to be multiplied by all combinations of other players in S which precede i and all possible combinations of remaining players which follow player i in the coalition. To arrive at the Shapley value for player i, divide the sum by the total number of combinations.

The Shapley value has some properties.

Efficiency The sum of Shapley values is equal to the value of a coalition formed by all players.

Symmetry The Shapley does not depend on the label of a player but only on its position in the characteristic function.

Linearity The Shapley value depends linearly on the values from the characteristic function v.

Dummy Axiom The Shapley value of a player who contributes nothing to any coalition is 0.

To produce Figure 3c and Figure 3d, we calculate the Shapley values of each factor in the comparison on the cumulative number of saved infections between the main scenario and the scenario without any of the factors for every day. Then, we divide up the saved infections on a particular day according to the Shapley values for the same day which yields the daily saved infections for each factor.

A.11 Overview Model Parameters

Table A.2. Contacts, Matching and Policies

name	notation	time active	source	description
n_contacts	$\eta_{c,\, n}$	always	Mossong *et al.* (*29*)	probability to have *n* contacts in contact type *c*. For non recurrent contacts the number of contacts is drawn for each individual every day. For recurrent contacts individuals are assigned group identifiers that determine the groups in which they meet either daily or weekly. The number of groups individuals belong to are calibrated to arrive at the number of recurrent contacts reported in Mossong *et al.* (*29*). See Section A.3.
degree_of_assortativity	$\alpha_{c,\, a_i,\, a_j,\, county_i,\, county_j}$	always	Mossong *et al.* (*29*)	probability in contact type *c* that an individual of age group a_i and county $county_i$ meets an individual of age group a_j and county $county_j$. Contacts can be assortative by age group, state and county. The degree of assortativity depends on the contact type *c*. See Section A.4 for details.
work_attend_multiplier	$\rho_{w,\, attend,\, t}$	always	Google, LLC (*47*)	share of workers that continue to have work contacts, i.e. do not work from home on date *t*. We use the reduction in work mobility reported by Google, LLC (*47*) as a proxy for the share of workers that work from home. See Section A.5 for details.
hygiene_multiplier	$\rho_{hygiene}$	since November 2020	estimated	reduction in transmission during work and educational contacts due to stricter hygiene measures such as wearing face masks. See Section A.9.
other_multiplier	$\rho_{other,\, t}$	always	estimated	reduction in the transmission during other contacts, such as leisure. This incorporates both hygiene measures as well as the reduction of physical meetings. There are nine breakpoints for the whole estimation period. See Section A.9 for details.

Table A.3. Infection Probabilities and Virus Variants

name	notation	time active	source	description
infection_prob	β_c	always	estimated	Base infection probability of contact type *c*. For each contact, this base probability is further adjusted by the seasonality, susceptibility of the contact and the virus strain. See Section A.9 for details.
susceptibility	ζ_a	always	Davies *et al.* (*1*)	Susceptibility to Covid-19 depends on a persons age group. The higher the age the more easily people become infected. The susceptibility of the oldest age group is normalized to one.
seasonality	κ_c	always	Gavenčiak *et al.* (*71*)	The probability to contract Covid-19 when exposed depends on the seasonality. Since different contact types are more or less subject to seasonal variation (e.g. by moving contacts outdoors) the seasonality also depends on the contact type. Refer to Section B.8 for an explanation.
variant_infectiousness	σ_v	always	Davies *et al.* (*2*)	Variant *v*'s infectiousness relative to the wild type.
variant_introduction	$\omega_{v,t}$	time dependent	estimated	Number of infections per 100,000 individuals of variant *v* to be introduced on day *t*. See Section A.9 for details.

Table A.4. Disease and Vaccination Model

name	notation	time active	source	description
p_duration_immune	$\gamma_{immune,\, d}$	always	see Section A.1	probability to stay immune for *d* days after having contracted CoViD-19
p_duration_until_infectious	$\gamma_{infectious,\, d}$	always	see Section A.1	probability to become infectious *d* days after infection
p_duration_of_infectiousness	$\gamma_{stop\ infectious,\, d}$	always	see Section A.1	probability that infectiousness lasts *d* days
p_duration_until_symptoms	$\gamma_{symptoms,\, a,\, d}$	always	see Section A.1	probability for individuals of age group *a* to develop symptoms *d* days (possibly infinite) after becoming infectious
p_duration_of_symptoms	$\gamma_{stop\ symptoms,\, d}$	always	see Section A.1	probability for individuals of age group *a* that symptoms last *d* days
p_duration_until_icu	$\gamma_{icu,\, a,\, d}$	always	see Section A.1	probability for symptomatic individuals of age group *a* to require intensive care *d* days (possibly infinite) after symptom onset
p_duration_of_icu	$\gamma_{stop\ icu,\, a,\, d}$	always	see Section A.1	probability to recover after *d* days from requiring intensive care
p_duration_until_death	$\gamma_{dead,\, a,\, d}$	always	see Section A.1	probability for individuals of age group *a* in intensive care to die after *d* days (possibly infinite)
p_until_immune_by_vaccine	$\gamma_{vacc,\, d}$	2021	see Section A.1	probability to develop immunity *d* days (possibly infinite) after being vaccinated
share_vaccine_refusers	ξ	always	Frisch (*72*)	share of individuals refusing to be vaccinated. See Section A.2

Table A.5. Rapid Testing

name	notation	time active	source	description
rapid_test_specificity	$p_{negative\|not\ infected}$	2021	Brümmer *et al.* (*73*)	the probability of an uninfected person to receive a negative rapid test result. See Section B.7.
rapid_test_sensitivity	$p_{positive\|infected,\ i,\ t}$	2021	see B.7	the probability of an infected person to receive a positive rapid test result. This depends on the timing of the test relative to the individuals onset of infectiousness. See Section B.7.
share_accepting_work_rapid_test_offer	$\pi_{w,\ d}$	2021	Betsch *et al.* (*48*)	share of workers that regularly pick up rapid test offers by their employers when they do not work from home. In our baseline specification this is time constant. See Section A.6.
share_workers_receiving_rapid_test_offer	$\pi_{w,\ s,\ t}$	2021	see A.6	share of workers that get a regular rapid test offer by their employer when they do not work from home on date *t*.
share_educ_workers_with_rapid_test	$\pi_{teacher,\ t}$	2021	see A.6	share of educational workers who test themselves as part of their work on date *t*
share_students_with_rapid_test	$\pi_{students,\ t}$	2021	see A.6	share of school pupils that do rapid tests at school on date *t*.
share_private_rapid_test_demand	$\pi_{private,\ t}$	2021	see A.6	share of individuals that do a rapid test when any of the private reason events such as a household member testing positive occur on date *t*.
rapid_test_educ_freq	$\theta_{t,\ educ}$	2021	see A.6	Frequency with which individuals test themselves in educational settings at time *t*. See Section A.6.
rapid_test_work_freq	$\theta_{t,\ work}$	2021	see A.6	Frequency with which complying workers test themselves at time *t*. See Section A.6.

Table A.6. PCR Testing, Case Detection and Behavioral Response

name	notation	time active	source	description
p_duration_until_test_result	$\gamma_{PCR,\,d}$	always	Robert Koch-Institut (*68*)	probability that it takes *d* days between the performance of a PCR test and receiving the result. See Section A.8.
share_of_tests_for_symptomatics	$\chi_{symptom,\,t}$	always	calibrated from RKI	share of positive PCR tests that are performed on individuals because of Covid-19 symptoms. See Section A.8.
share_w_positive_rapid_test_requesting_pcr	$\chi_{confirmation}$	2021	Betsch *et al.* (*48*)	share of individuals with positive rapid test that seek a PCR test. See Section A.8.
share_known_cases_without_rapid_tests	ψ_t	always	Paul *et al.* (*65*)	share of cases that would be detected in the absence of rapid tests (see Section B.7)
symptomatic_multiplier	$\tau_{symptoms,\,c}$	always	see A.8	share of symptomatic individuals that still have contacts of type *c*.
positive_pcr_multiplier	$\tau_{positive\ PCR,\,c}$	always	see A.8	share of individuals with a positive PCR test that still have contacts of type *c*.
positive_rapid_test_multiplier	$\tau_{positive\ rapid\ test,\,c}$	2021	Betsch *et al.* (*48*)	share of individuals with a recent positive rapid test that still have contacts of type *c*. See Section A.8

A.12 Reproducibility

The source code used for this paper is open source and available under the MIT License. It is split into two parts

- The source code for the model can be found at https://github.com/covid-19-impact-lab/sid/ and its documentation at https://sid-dev.readthedocs.io.
- The source code for the application to Germany can be found at https://github.com/covid-19-impact-lab/sid-germany/ with a shorter documentation at https://sid-germany.readthedocs.io.

We are grateful to the authors and contributors of the following software packages upon which our software is built: conda (*74*), conda-forge (*75*) dask (*76*), estimagic (*77*), holoviews (*78*), matplotlib (*79*), numba (*80*), numpy (*81*), pandas (*82*, *83*), pytask (*84*), Python (*85*), scipy (*86*), and seaborn (*87*).

Appendix B: Supplementary Text

B.1 Literature Review

A commonly used model class in epidemiology are agent-based simulation models. In a prototypical agent-based simulation model, individuals are simulated as moving particles. Infections take place when two particles come closer than a certain contact radius (e.g. Silva *et al.* (*88*) and Cuevas (*89*)). While the simulation approach makes it easy to incorporate heterogeneity in disease progression, it is hard to incorporate heterogeneity in meeting patterns. Moreover, policies are modeled as changes in the contact radius or momentum equation of the particles. The translation from real policies to corresponding model parameters is a hard task.

These shortcomings have motivated variations of agent-based simulation models where moving particles have been replaced by contact networks for households, work and random contacts. The OpenABM-Covid-19 model by Hinch *et al.* (*90*) and the model by Aleta *et al.* (*91*) are the closest in spirit to ours.

The OpenABM-Covid-19 model by Hinch *et al.* (*90*) also uses detailed contact networks for workplaces, schools and households and can evaluate the effect of several NPIs. The main focus of their application are contact tracing policies (*92*). Recently they have also added support for multiple virus strains and vaccinations.

Aleta *et al.* (*91*) develop an agent-based simulation model with a very high geographical resolution by estimating contact networks from fine grained mobility data for the Boston metropolitan area. They use this model to show how NPIs, contact tracing and PCR testing can influence the infection dynamics. However, they do not calibrate their model to match actual infection numbers which makes it more suitable to explore the general mechanics of different disease mitigation measures than for their quantitative evaluation.

Bicher *et al.* (*93*) simulate the entire Austrian population. They use data from the first wave (February 21 to April 9, 2020) to calibrate their model and predict the effect of different NPIs and contact tracing policies until November 2020. They use the same data provided by Mossong *et al.* (*29*) as we to calibrate contact networks for households, workplaces and schools. The model focuses on analyzing the effect of different contact tracing strategies and not on modelling enacted Austrian policies over a long period of time.

Moreover, there are several working papers that develop agent-based simulation models with contact networks in conjunction with economic models. Examples are Basurto *et al.* (*94*), Delli Gatti and Reissl (*95*) and Mellacher (*96*).

Our model combines elements from the above models and adds several others. To the best of our knowledge, our model is the only one with the following features:

1. The free model parameters have been estimated with the method of simulated moments (*69*). Despite having few free parameters our model does an excellent job in explaining observed case numbers and the spread of the B.1.1.7 mutation over more than nine months of data.

2. We have an extremely fine grained representation of schools and preschools. We can thus easily model all schooling policies that have been implemented in Germany in the past nine months. This includes complete school closures, phases where only those students whose parents could not find any private childcare arrangement could attend, split class approaches for some or all age groups and combinations thereof. Moreover, we can account for additional hygiene measures whose effect is estimated inside the model.

3. We model the evolution of the pandemic and all enacted policies since the start of the second wave. Since the vast majority of cases has occurred in that time period and we also model unobserved infections our simulations take into account that many people are already immune because they have recovered from an infection and that this immunity is not spread randomly across the population.

4. We have an extremely detailed model of PCR and rapid tests with a share of detected cases that varies over time and across age groups.

5. Our model is designed to combine information from many different data sources. Examples are surveys on rapid test demand (*48*), reaction to test results (*48*), contact diaries (*29*), share of detected cases (*65*) and many more.

B.2 Summary

We use an agent-based simulation model with detailed contact networks. The model structure is depicted in Figure 1a.

We distinguish between eight types of contacts which are all listed in Figure 1a: households, recurrent and random work contacts, recurrent and random leisure contacts, as well as nursery, preschool, and school contacts.

The number of contacts is translated into infections by a matching algorithm. There are different matching algorithms for recurrent contacts (e.g. classmates, family members) and non-recurrent contacts (e.g. clients, contacts in supermarkets). All types of contacts can be assortative with respect to geographic and demographic characteristics.

The infection probabilities of contacts vary with contact type, age of the susceptible person, and the virus strain of the infected person. Moreover, they follow a seasonal pattern. The strength of the seasonality effect is higher for contacts that are easy to be moved to an outside location in summer (such as leisure contacts) and smaller for contacts that take place inside even in summer (e.g. work contacts).

Once a person is infected, the disease progresses in a fairly standard way which is depicted in Figure 1b. Asymptomatic cases and cases with mild symptoms are infectious for some time and recover eventually. Cases with severe symptoms additionally require hospitalization and lead to either recovery or death.

After rapid tests become available, people who work or go to school can receive rapid tests there. Moreover, people can decide to do a rapid test if they develop symptoms, have many planned contacts or have a sick or positively tested household member. People who have a positive rapid test demand a confirmatory PCR test with a certain probability. Moreover, PCR tests can be demanded because of symptoms or randomly.

This rich model of PCR and rapid tests leads to a share of detected cases that varies over time and across age groups. It also allows to quantify the effect of changes in testing policies on the dynamic of infections.

People who have symptoms or received a positive test can reduce their number of contacts across all contact types endogenously. The extent to which this is done is calibrated from survey data.

The model makes it very simple to translate policies into model quantities. For example, school closures imply the complete suspension of school contacts. A strict lockdown implies shutting down work contacts of all people who are not employed in a systemically relevant sector. It is also possible to have more sophisticated policies that condition the number of contacts on observable characteristics, risk contacts or health states.

An important feature of the model is that the number of contacts an individual has of each contact type can be calibrated from publicly available data (*29*). This in turn allows us to estimate policy-invariant infection probabilities from time series of infection and death rates using the method of simulated moments (*69*). Since the infection probabilities are time-invariant, data collected since the beginning of the pandemic can be used for estimation. Moreover, since we model the testing strategies that were in place at each point in time, we can correct the estimates for the fact that not all infections are observed.

The model has a very modular structure and can easily be extended to distinguish more contact types, add more stages to the disease progression, implement

new policies or test demand models. The main bottleneck is not complexity or computational cost but the availability of data to calibrate additional model features.

B.3 Modeling Numbers of Contacts

Consider a hypothetical population of 1,000 individuals in which 50 were infected with a novel infectious disease. From this alone, it is impossible to say whether only those 50 people had contact with an infectious person and the disease has an infection probability per contact (β) of one or whether everyone met one infectious person but the disease has an infection probability of only 5 percent per contact. SEIR models do not distinguish between the number of contacts (η) and the infectiousness of each contact (β). Instead, they combine the two into one parameter that is not invariant to social distancing policies.

To model social distancing policies, we need to disentangle the effects of the number of contacts of each individual and the effect of mostly policy-invariant infection probabilities specific to each contact type.

The number and type of contacts in our model can be easily extended. Each type of contacts is described by a function that maps individual characteristics, health states and the date into a number of planned contacts for each individual. This allows to model a wide range of contact types.

In our empirical application we distinguish the following contact types that are depicted in Figure 1a and can be further grouped in the categories household, work, education and others:

- Households: Each household member meets all other household members every day.
- Recurrent work contacts: These capture contacts with coworkers, repeating clients and superiors. Some of these recurrent contacts take place on every workday, others just once per week. The contacts are assortative in geographical location and age.
- Non recurrent work contacts: Working adults have contacts with randomly drawn other people, which are assortative in geographical location and age.
- Schools: Each student meets all of his classmates every day. Class sizes are calibrated to be representative for Germany and students have the same age and mostly live in the same county. Schools are closed on weekends and during vacations, which vary by states. School classes also meet six teachers every day and some of the teachers meet each other.
- Preschools: Children who are between three and six years old attend preschool. Each group consists of nine children of mixed ages and two adults who live mostly in the same county. They all meet each other every work day when there are no vacations.

- Nurseries: Children younger than three years may attend a nursery and interact with one adult. The age of the children varies within groups but all live in the same county. They all meet each other every work day when there are no vacations.
- Non recurrent other contacts: Contacts with randomly drawn other people, which are assortative with respect to geographic location and and age group. This contact type reflects contacts during leisure activities, grocery shopping, medical appointments, etc..
- Recurrent other contacts representing contacts with friends neighbours or family members who do not live in the same household. Some of these contacts happen daily, others only once per week. They are assortative in geographic location and age.

The number of random and recurrent contacts at the workplace, during leisure activities and at home is calibrated with data provided by Mossong *et al.* (*29*). For details see Section A.3. In particular, we sample the number of contacts or group sizes from empirical distributions. It would also be possible to use economic or other behavioral models to predict the number of contacts.

B.4 Reducing Numbers of Contacts via NPIs

Our model makes it very easy to model a wide range of NPIs, either in isolation or simultaneously. This is important for two reasons: Firstly, it allows to predict and quantify the effect of novel NPIs. Secondly, it allows to model the actually implemented policy environment in great detail, which is necessary to use the full time series of infections and fatality rates to estimate the model parameters.[12]

Instead of thinking of policies as completely replacing how many contacts people have, it is often more helpful to think of them as adjusting the pre-pandemic number of contacts. Therefore, we implement policies as a step that happens after the number of contacts is calculated but before individuals are matched.

On an abstract level, a policy is a functions that modifies the number of contacts of one contact type. This function can be random or deterministic. For example, school closures simply set all school contacts to zero. A work from home mandate leads to a share of workers staying home every day whereas those who cannot work from home are unaffected. Hygiene measures at work randomly reduce the number of infectious contacts for all workers who still go to work.

Policies can also interact. For example, school vacations are temporally reducing school contacts to zero while at the same time increasing other contacts to account for increased leisure activities and family visits during this time. This is important to

12. See Avery *et al.* (*97*) for an explanation why it can be harmful to use too long time series to estimate simple SEIR type models.

reproduce the finding that school vacations do not reduce infection numbers even though schools lead to infections when open (*98*).

The most complex policies are typically found in the education sector. Since the beginning of 2021 schools have switched back and fourth between full closures, split class approaches with alternating schedules for some or all age groups and reopening while maintaining hygiene measures. On top of that there are different policies for allowing young students whose parents work full time to attend school even on days where they normally would not. For details on how we calibrate these policies see Section A.5.

Importantly, policies can depend on the health states of participating individuals. For example children rarely go to school when they have symptoms. It would even be possible to quarantine entire school classes if one student tested positive and many other forms of contact tracing. For an application of our model showcasing private contact tracing in the context of the Christmas holidays see (*99*).

Not all things that reduce contacts compared to the pre-pandemic level are driven by NPIs. Therefore, we also model endogenous contact reductions that depend on the health state of individuals. Other possible factors could include things such as the local incidence. The extent to which contacts are reduced can be calibrated from surveys.

B.5 Matching Individuals

The empirical data described above only allows to estimate the number of contacts each person has. In order to simulate transmissions of Covid-19, the numbers of contacts have to be translated into actual meetings between people. This is achieved by a matching algorithm:

As described in section B.3, some contact types are recurrent (i.e. the same people meet regularly), others are non-recurrent (i.e. it would only be by accident that two people meet twice). The matching process is different for recurrent and non recurrent contact models.

Recurrent contacts are described by two components: 1) A set of time invariant groups, such as school classes or groups of co-workers. Those groups are generated once at the beginning of the simulation. The groups can be sampled from empirical data or created by randomly matching simulated individuals into groups. 2) A deterministic or random function that takes the value 0 (non-participating) and 1 (participating) and can depend on the weekday, date and health states of the entire population. This can be used to model things like vacations, weekends or symptomatic people who stay home (see section B.4 for details).

Given those two components, the disease transmission for recurrent contacts is extremely simple: On each simulated day, every person who does not stay home meets all other group members who do not stay home. If there is a contact between individual i who is infected with virus variant v and infectious and individual j who

is in age group a and susceptible, then j becomes infected with the following probability

$$P(infection) = \beta_c \cdot s_{c,t} \cdot \sigma_v \cdot \zeta_a \tag{B.1}$$

where β_c denotes the base infection probability of contact type c, $s_{c,t}$ is a seasonality factor between zero and one that depends on the contact type c and time t (see Equation B.5), σ_v is the infectiousness factor of virus variant v and ζ_a is an age dependent susceptibility factor.

The assumption that all group members have contacts with all other group members is not fully realistic, but a good approximation to reality, especially in light of the suspected role of aerosol transmission for Covid-19 (*100*, *101*). Alternatively, the infection probability of recurrent contact types can be interpreted as being the product of a true infection probability and the probability that an actual contact takes place.

The matching of non-recurrent contact types is more difficult because the contact network is resampled randomly every day. Moreover, it needs to allow for assortative matching. In our application, all random contacts are assortative with respect to age group a (it is usually more likely to meet people from the same age group) and county (it is more likely to meet people from the same county) but in principle any set of discrete variables can be used. This set of variables that influence matching probabilities induce a discrete partition of the population into groups.

Below we first describe one iteration of a simplified matching algorithm that illustrates what we want to achieve. In practice, we approximate the result of this matching algorithm by a two stage sampling procedure that is computationally more efficient. The matching is done for each non-recurrent contact type c. The following step is repeated until no individual has unmatched contacts left. Let z be an iteration counter for the matching algorithm and i denote the individual whose unmatched contacts we are trying to match.

Let $K_{z,i,c}$ denote the number of unmatched contacts of individual i of contact type c before iteration z is completed. Note that $K_{z,i,c} \leq n_{ic}$ which is the total number of contacts individual i has of type c.

Let a_i denote i's age group and $county_i$ her county of residence.

We first draw individual j from the distribution defined by probability mass function F_z over individuals $j \neq i$ in the synthetic population where the probability f_{zj} is calculated as follows:

$$f_{zj} = \underbrace{\alpha_{c,a_i,a_j,county_i,county_j}}_{\text{Group Probability}} \cdot \underbrace{\frac{K_{z,j,c}}{\sum_{l=1}^{N} K_{z,l,c} \cdot \mathbb{I}_{county_l = county_j \wedge a_l = a_j}}}_{\text{Individual Probability}} \tag{B.2}$$

We then draw an individual j. If one of the two participants is susceptible and the other one is infectious, we sample whether an infection takes place. The success

probability for this event is calculated as in Equation B.1. Finally we update the remaining numbers of unmatched contacts by setting:

$$K_{z+1,i,c} = K_{z,i,c} - 1 \tag{B.3}$$

$$K_{z+1,j,c} = K_{z,j,c} - 1 \tag{B.4}$$

The runtime of this algorithm scales roughly cubic in the number N of simulated individuals. This is because the number of iterations is proportional to N, in each iteration we have evaluate Equation B.2 N times and each evaluation of that equation entails a sum over N individuals.

This makes it prohibitively expensive. We therefore replace the above algorithm by a two stage sampling procedure, where we first sample the group from which individual j will be drawn according to the group probabilities defined in Equation B.2. Next we sample an individual from this group with the Individual probabilities defined in Equation B.2.

Thus, while the calculation of any given second stage probability entails exactly the same number of calculations as before we do not have to calculate a second stage probability for all simulated individuals but only for those who are members of the group that was sampled in the first stage.

It is easy to see that ex-ante the probability of being sampled are identical between the two stage sampling and the one stage sampling. The only drawback is that towards the end of the matching process it becomes possible to sample a group in which no unmatched contacts are left. In our empirical application this happens extremely rarely. This is so for two reasons: Firstly, the first stage sampling probabilities have been estimated from the same dataset as the numbers of contacts so there cannot be any mismatches such as for example a group that has a low probability of being sampled in the first stage but where all members have many contacts. Secondly, the group sizes are relatively large and we go over individuals in random order. Therefore, groups where no unmatched contacts remain only occur very late in the matching process.[13]

B.6 Course of Disease

The disease progression in the model is fairly standard. It is depicted in Figure 1b and the values and source of the relevant parameters are described in Section A.1.

First, infected individuals will become infectious after one to five days. Overall, about one third of people remain asymptomatic. The rest develop symptoms about one to two days after they become infectious. Modeling asymptomatic and

13. If unmatched contacts were a concern one could simply use the fast two stage sampling process for a first pass over contacts and then match all remaining contacts with the slow algorithm.

pre-symptomatic cases is important because those people do not reduce their contacts nor do they have an elevated probability to demand a test. Thus they can potentially infect many other people (*102*). The probability to develop symptoms with Covid-19 is highly age dependent with 75% of children not developing clinical symptoms (*1*).

A small share of symptomatic people will develop strong symptoms that require intensive care. The exact share and time span is age-dependent. An age-dependent share of intensive care unit (ICU) patients will die after spending up to 32 days in intensive care. Moreover, if the ICU capacity was reached, all patients who require intensive care but do not receive it die.

We allow the progression of the disease to be stochastic in two ways: Firstly, state changes only occur with a certain probability (e.g. only a fraction of infected individuals develops symptoms). Secondly, the number of periods for which an individual remains in a state is drawn randomly. The parameters that govern these processes are taken from the literature and detailed in Section A.1. For an overview of our disease progression parameters see Table A.4.

B.7 Testing

Having a realistic model of PCR and rapid tests is crucial for two reasons: Firstly, only via a testing model can the simulated infections from the model be made comparable to official case numbers. Secondly, individuals with undetected or not yet detected infections are an important driver of the pandemic.

In principle, our modeling approach is flexible enough to incorporate mechanistic test demand, allocation and processing models. However, there is not enough data available to calibrate such a mechanistic model.

Therefore, we build a simpler model of PCR and rapid tests that can be calibrated with available data on test demand and availability and – nevertheless – can produce a share of undetected cases that varies over time and across age groups and agrees with other estimates over the time periods where they are available.

PCR tests are modeled since the beginning of the simulation and determine whether a infection is officially recorded. Rapid tests are only added at the beginning of 2021. Positive rapid tests do not enter official case numbers directly, but most people with a positive rapid tests demand a confirmatory PCR test. However, positive rapid tests can have a strong effect on the infection dynamics because they trigger contact reductions and additional rapid tests.

During 2020 people can demand PCR tests either because they have symptoms or randomly. The probability that a PCR test is performed in each of the two situations depends on the number of new infections and the number of available tests. Thus, it varies strongly over time and is unknown.

To distribute the correct number of PCR tests among symptomatic and asymptomatic infections without knowing explicit test demand probabilities, we use the

following approach: First, we calculate the total number of positive PCR tests by multiplying the number of newly infected individuals with an estimate of the share of detected cases from the Dunkelzifferradar project (*65*). Next, we determine how many of these tests should go to symptomatic and asymptomatic individuals from data by the RKI (*68*). Then, we sample the individuals to which those tests are allocated from the pools of symptomatic and asymptomatic infected but not yet tested individuals.

Sampling uniformly from the pool of symptomatic individuals ensures that age groups who are more likely to develop symptoms are also more likely to receive tests. Thus, the share of detected cases is much higher for the elderly than for children in time periods where many tests are done because of symptoms.

At the beginning of 2021, two challenges arise: Firstly, the externally estimated share of detected cases from Dunkelzifferradar project (*65*) can no longer be used because it is based on the case fatality rate which changes drastically due to vaccinations. Secondly, rapid tests become available at a large scale.

We solve the first challenge by assuming that the share of detected cases would have remained at the level it reached before Christmas if rapid tests had not become available. While this is only an approximation to reality, changes in the share of detected cases that would have happened without rapid tests are very likely to be small compared to the changes caused by rapid tests.

The second challenge is solved by mechanistic rapid test demand models for the workplace, schools and by private individuals. The calibration of these models is described in Section A.6. Figure 2d shows that the number of performed rapid tests in the model fits the empirical data well (where empirical data is available).

In contrast to PCR tests, rapid tests are not perfect and can be falsely positive or falsely negative. While the specificity of rapid tests is calibrated at 99.4% (*73*), their sensitivity strongly depends on the timing of the rapid test relative to the start of infectiousness. We follow (*103*) for our main results: Before the onset of infectiousness the sensitivity is very low (35%). On the first day of infectiousness it is much higher (88%) but still lower than during the remaining infectious period (92%). After infectiousness stops, the sensitivity drops to 50%. We show that our results are robust to more conservative assumptions in Section B.12.

Modeling the diagnostic gap before and at the beginning of infectiousness is very important to address concerns that rapid tests are too unreliable to serve as screening devices.

We do not distinguish between self administered rapid tests and those that are administered by medical personnel. While there were concerns that self administered tests are less reliable, a recent study has found no basis for this concern (*104*).

While rapid tests do not directly enter official case numbers, 82% ($\chi_{confirmation}$) of positively tested individuals seek a PCR test (*48*). Importantly, those PCR tests are made in addition to the tests that would have been done otherwise. Section B.13 discusses the effect of rapid tests on the share of detected cases.

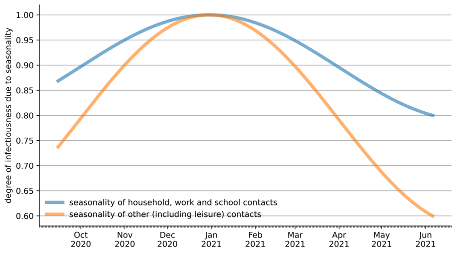

Figure B.1. Seasonality by Type of Contact

Note: We model seasonality as a factor that reduces the probability of infection of all encounters. The factor depends on the day and is calculated from a sinus shaped function with its maximum on January 1. Since seasonality can affect the transmission both through physical conditions such as temperature and humidity as well as through the numbers of contacts that take place outside we assume two seasonality factors. One for other contacts which we expect to be strongly affected by fairer weather with a maximum reduction of 42% in the infection probability. The other seasonality only makes contacts up to 21% less infectious and is applied to household, work and school contacts.

B.8 Seasonality

It is widely acknowledged that the transmission of SARS-CoV-2 is subject to seasonal influences. Infectiousness is increased in winter when most contacts take place inside and the immune system is weakened by low levels of vitamin D, dry air and large temperature swings. For a detailed overview of possible drivers see (*105*).

We follow (*106*) and (*71*) in modeling seasonality in the transmission of SARS-CoV-2 as a multiplicative factor on infection probabilities. The factor follows a sine curve that reaches its maximum at January 1 and its minimum on June 30.

For simplicity we normalize the factor to reach one at its maximum. Thus, the formula of the seasonality factor is given by:

$$s_{c,t} \;=\; 1 + 0.5\kappa_c sin\left(\pi\left(\frac{1}{2} + \frac{t}{182.5}\right)\right) - 0.5\kappa_c \tag{B.5}$$

Where κ_c is difference in the seasonality factor between peak infectiousness and lowest infectiousness.

The subscript c is needed because the strength of the seasonality effect differs across contact types: Work, household and school contacts are likely to take place inside even in summer. Thus they are only subject to seasonality due to factors that influence the immune system. Other contacts (for example meeting friends and while doing leisure activities) are mostly happening outside in the summer. Therefore, transmission via those contacts should have a stronger seasonal pattern.

We calibrate κ_{strong} to 0.42 and κ_{weak} to 0.21. This is in line with (*71*) and (*106*).

The two seasonality curves are shown in Figure B.1.

B.9 Initial Conditions

Consider a situation where you want to start a simulation with the beginning set amidst the pandemic. It means that several thousands of individuals should already have recovered from the disease, be infectious, symptomatic or in intensive care at the start of your simulation. Additionally, the sample of infectious people who will determine the course of the pandemic in the following periods is likely not representative of the whole population because of differences in behavior (number of contacts, assortativity), past policies (school closures), etc.. The distribution of health states in the population at the beginning of the simulation is called initial conditions.

To come up with realistic initial conditions, we match reported infections from official data to simulated individuals by age group and county. We use one month of data to generate initial conditions with in all possible health states. Meanwhile health states evolve until the beginning of the simulation period without simulating infections by contacts. We also correct reported infections for a reporting lag and scale them up by the share of detected cases to arrive at the true number of infections.

B.10 Model Fit

This section compares simulated data from our model with empirical data from Germany. We look at observed infections (overall as well as by age group and federal state), the effective replication number, the spread of B.1.1.7 and vaccinations.

Overall, our model achieves an excellent fit of the two waves of infections with few free parameters (Figure B.2a). As a result the effective replication number R_t also closely follows that reported by the RKI (see Figure B.2b). We also achieve an excellent fit for most age groups in Germany. The fit is also good for many German federal states. Despite the fact that the number of performed rapid tests and their distribution in the population are determined endogenously in our model, we fit the share of the population with at least a weekly rapid test very well. For the share of individuals who have ever done a rapid test we err on the side of too few test.

Our fit of the infection rates in Germany between October 2020 and June 2021 is excellent. The incidence in our model matches both the levels and the shape of the reported incidence almost perfectly. When the prevalence of the virus is high and especially after explosive growth phases, the effect of random events on the incidence is large. Therefore all reported simulations average over at least 30 simulation runs which is enough to reduce the sampling uncertainty to a negligible level.

Our fit of the effective replication number R_t closely follows the values reported by the RKI (see Figure B.2b) even though we calculate R_t on all infected individuals not just the detected cases. This explains why the R_t in our simulations is higher during phases where the share of detected cases (ψ_t) falls. This is the case in the

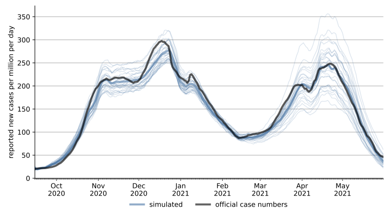

(a) Observed Incidence in the Model and as Reported by the RKI

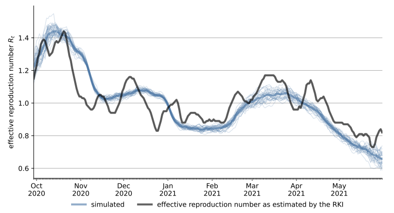

(b) Effective Replication Number R_t in the Model and as Reported by the RKI

Figure B.2. Model Fit of the Reported Cases and the Effective Replication Number

Note: Both figures show averages and single runs. The average is the thick line. Single runs are shown as lighter and thinner lines. We averaged and show 30 simulation runs. The left figure shows the daily incidence rate per million for the simulated reported infection rates. The official case numbers as reported by the RKI are plotted in black. The fit is overall very good. The right figure shows the effective replication number (R_t) as reported by the RKI and as calculated in our model. The R_t gives the average number of new infections caused by one infected individual. The R_t in our model broadly follows the R_t reported by the RKI. Two differences stand out. Firstly, the RKI's R_t drops faster in November. This is likely due to a decline in the estimated overall share of detected cases (ψ_t) when the second wave hit Germany. The second difference is from mid February to mid March where the RKI's reported R_t increased more rapidly than that in our model. Here the opposite effect can be expected. During this time rapid tests increased strongly leading to more cases being detected. In the short term this leads an R_t estimation that is based on detected cases to overestimate the replication number. For legibility reasons, all lines are rolling 7-day averages.

fall of 2020 (see Figure A.7) where the RKI underestimated the effective replication number due to observing a falling share of cases. Analogously, the R_t in our simulations is lower than the R_t reported by the RKI in spring where the share of known cases increased due to increased rapid testing.

Zooming into the different age groups in Figure B.3, we can see that our model is also able to reproduce the infection rates on this level. The only major deviation from this pattern is that our model predicts too few infections for the 80 to 100 year olds. This was to be expected because our synthetic population does not include inhabitants of nursing homes. Outbreaks in nursing homes led to a large number of infections among the oldest during the second wave of the pandemic in Germany. Moreover, the model predicts too few observed infections for the 15 to 34 years old at the end of 2020 and the 5 to 14 years old in April and May 2021. The former is likely due to the fact that this age group has a very active social life which is not fully captured by our contact networks. The latter probably comes from a too conservative model of school reopenings.

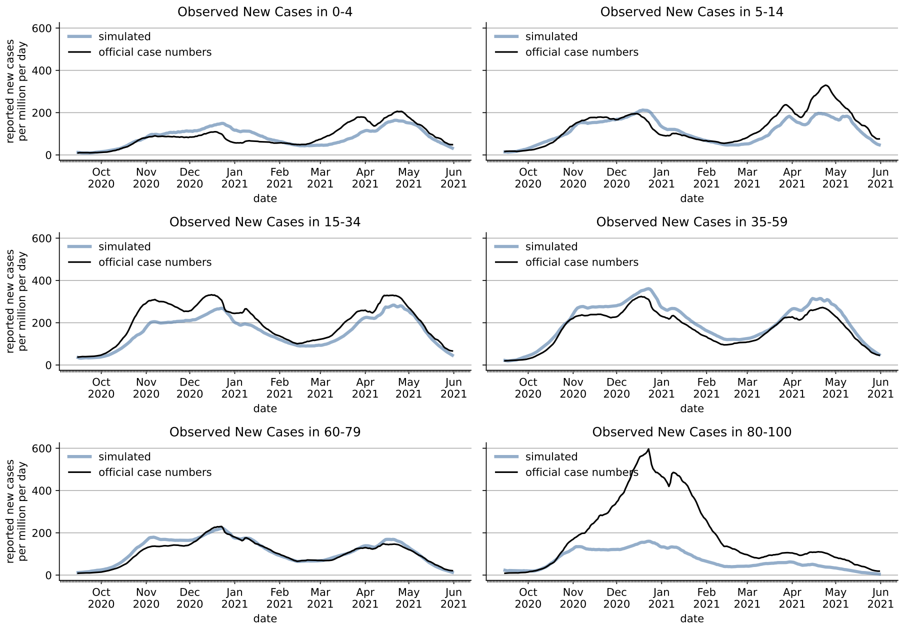

Figure B.3. Simulated and Empirical Infections by Age Group

Note: The figure shows the number of reported versus simulated cases per one million people per day for different age groups. The age group of individuals above 80 needs to be interpreted with caution because our synthetic population only includes private households, i.e. nursing homes are not represented in our model. They accounted for many cases and deaths in the winter of 2020 and many 80 to 100 year olds live in these facilities. We average over 30 simulation runs. For legibility reasons, all lines are rolling 7-day averages.

Our model fit is also very good for the different German federal states. This holds not only for the large states such as North Rhine-Westphalia or Bavaria but also for many smaller states such as Hessen or Rhineland-Palatinate. This shows that using school vacations dates and work mobility reductions by (*47*) at the state level combined with county and age group specific initial conditions (see Section B.9) and county level assortativity of contacts is sufficient to represent many local differences. The fit is especially good given that our model does not aim to have a high local resolution. For example we abstract from population density and cross-border travel. It is, thus, unsurprising that there are states that we do not match well, such as very thinly populated Mecklenburg-Vorpommern and Schleswig-Holstein or Saxony with its large border to the Czech Republic that had a much higher incidence than Germany.

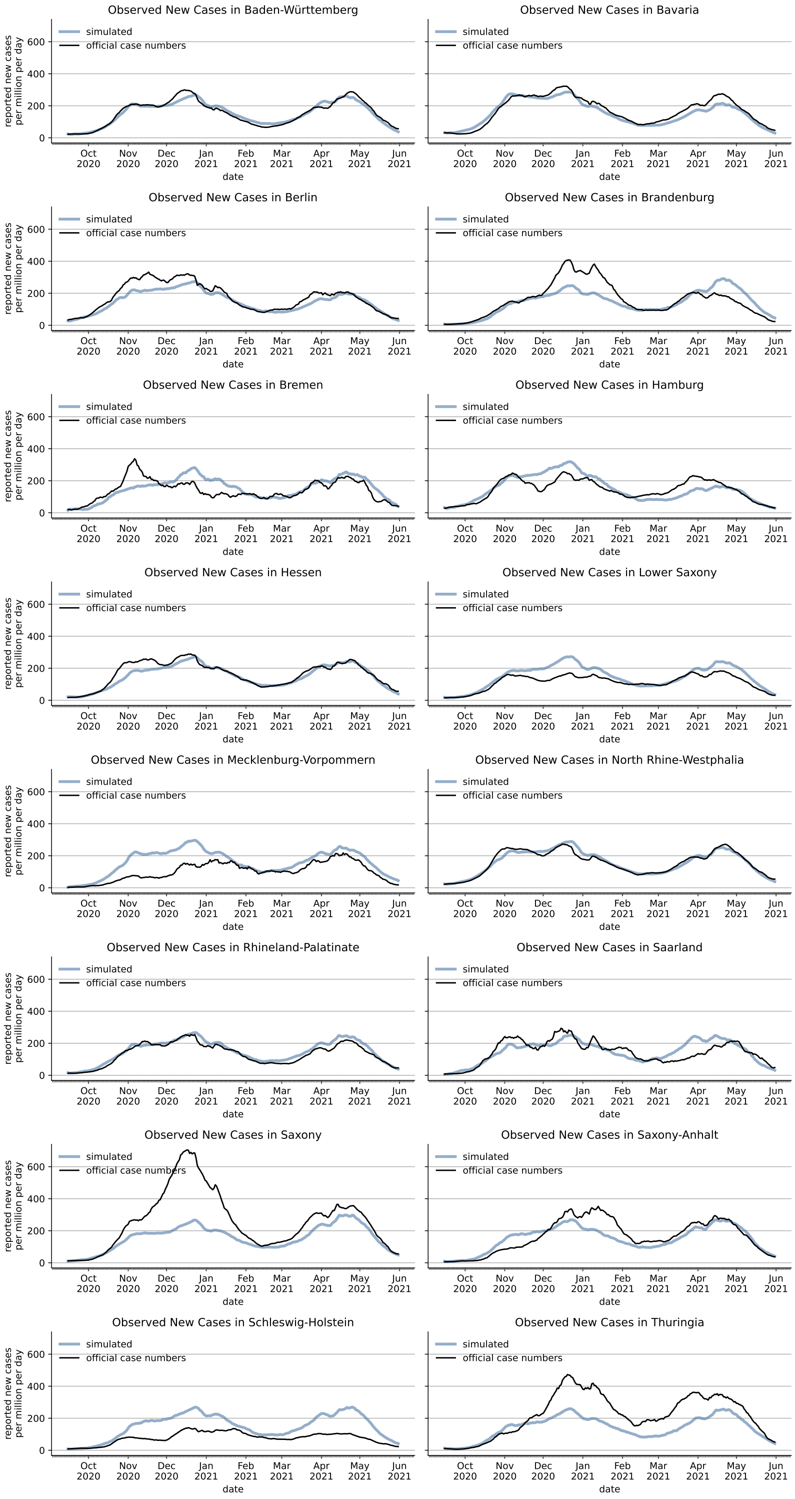

Figure B.4. Simulated and Empirical Infections by Federal State

Note: The figure shows the number of reported versus simulated cases per one million people per day for different federal states. We averaged over 30 simulation runs. For legibility reasons, all lines are rolling 7-day averages.

We fit the proliferation of the B.1.1.7 variant quite exactly despite only introducing a few cases in January ($\omega_{B.1.1.7,t}$) as can be seen in Figure B.5a. Since we only model B.1.1.7 and do not include other variants, B.1.1.7 reaches a share of nearly 100% by May while the true rate plateaued at 90%. By the end of May B.1.617.2 gained traction in Germany. However, given that B.1.617.2 made up less than 5% even at the end of our simulation period, we did not include it in our model.

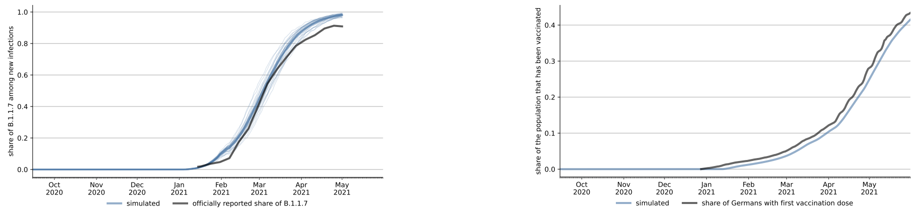

(a) Share of B.1.1.7 in the Model and as Reported by the RKI

(b) Share of Vaccinated Individuals in the Model and the German Population

Figure B.5. Model Fit of the Share of B.1.1.7 and Vaccinations

Note: The left figure shows the share of B.1.1.7 as reported by the RKI and as calculated in our model. We only introduce a few cases over the course of January. From then B.1.1.7 takes over endogenously through its increased infectiousness ($\sigma_{B.1.1.7}$). The right figure shows the rate of individuals that are vaccinated in our synthetic population versus in the general German population. For legibility reasons, all lines are rolling 7-day averages.

The fit of the share of vaccinated individuals can be seen in Figure B.5b. In Germany, vaccines were rolled out according to four priority groups. The first vaccines were mostly reserved for nursing homes and some selected professions such as first responders. Since we do not have nursing home inhabitants in our model, we subtract the first percent of vaccinations which is equivalent to the share of Germans living in nursing homes. Afterwards, the share of vaccinated individuals in the population follows the German increase exactly. We took great care to model the prioritization of older individuals and professions that cannot reduce physical contact easily such as teachers or medical staff (see Section A.2 and Figure A.1 for the vaccination rates in our model by age group).

The most difficult moment to match in our model is the rapid test demand. This is because we have five different channels through which individuals demand rapid tests and many of the demand curves are at least partially calibrated through survey data. It is therefore very reassuring that we fit the share of individuals that do weekly rapid tests almost perfectly. For the share of individuals that have ever done a rapid test our model is conservative. There are two reasons for this: Firstly, we do not model people who have done rapid tests out of curiosity once they became available. Secondly, in the model, the decision to take a rapid test is based on a time invariant individual specific compliance factor without any additional random components. While this captures important features of rapid test demand it abstracts from people who turn down rapid tests most of the time but accept them sometimes.

Fortunately, Section B.11 shows that our main results are robust to changes in the exact shares of individuals demanding rapid tests.

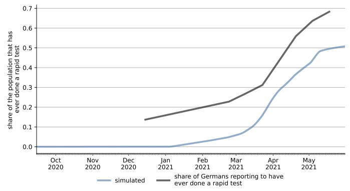

(a) Share of the Population That Has Ever Done a Rapid Test

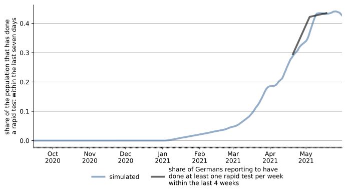

(b) Share of the Population That Did a Rapid Test in the Last Week

Figure B.6. Share of Individuals With Rapid Tests

Note: Both panels compare empirical and simulated rapid test demands. The empirical data comes from Betsch *et al.* (*107*). The left panel compares the share of individuals who have ever done a rapid test. The right panel compares the share of individuals who have done a rapid test within the last seven days in our simulation compared to the share reporting to have done at least weekly rapid tests in the last four weeks in the COSMO survey. Overall our calibration of rapid tests are slightly conservative. The overall share is below that in the study. We fit the share of weekly tests quite exactly. For legibility reasons, all simulated lines are rolling 7-day averages.

B.11 Model Validation

Achieving a good in-sample fit does not necessarily guarantee that our model will also be able to make out of sample predictions. For example, it could be that the results are very sensitive to the exact number of vaccinations, the work mobility multiplier ($\rho_{w,\,attend,\,t}$) or the number of performed rapid tests (governed by the π parameters) – all of which are things that cannot be known exactly ex-ante.

In this section we compare simulated infections that use all available data with out of sample predictions that only use data that was available at March 1 2021.

For the out of sample predictions we predict the number of vaccinations between March and June with a simple linear regression model that was fitted on vaccine data from February. This prediction model is pessimistic compared to the actual number of vaccinations. The work mobility multiplier ($\rho_{w,\,attend,\,t}$) is predicted to be constant at a value of 0.75, which is an approximate average of the second half of February. This turned out to be optimistic.

The area that is fraught with the most uncertainty is the introduction of rapid tests, because it comprises both supply and demand factors. Moreover, accurately predicting the number of rapid tests is expected to be important because rapid tests play a large role for the transmission dynamic.

We therefore make a scenario analysis with different assumptions on the availability of rapid tests. The number of rapid tests performed in each scenario can be seen in Figure B.7. All scenarios are the same until March 1 and have the same level of rapid tests when all supply constraints are resolved. They differ in the date

at which the full number of tests is reached. For students ($\pi_{students,\, t}$) and teachers ($\pi_{teacher,\, t}$) the full number of rapid tests is reached after the Easter holidays in all scenarios. For rapid tests in the workplace ($\pi_{w,\, s,\, t}$) and private rapid tests ($\pi_{private,\, t}$) it is reached between May 1 and June 10, depending on the scenario.

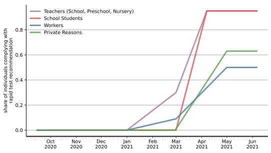

(a) Rapid Test Parameters: Early Scenario

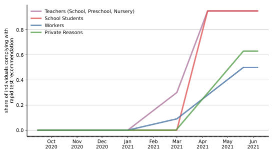

(b) Rapid Test Parameters: Medium Scenario

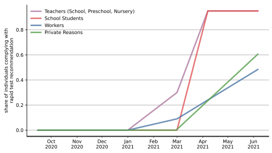

(c) Rapid Test Parameters: Late Scenario

Figure B.7. Rapid Test Introduction in the Three Scenarios

Note: Number of rapid tests performed in the different prediction scenarios. All scenarios are the same until March 1 and have the same level of rapid tests when all supply constraints are resolved. They differ in the date at which the full number of tests is reached. For students ($\pi_{students,\, t}$) and teachers ($\pi_{teacher,\, t}$) the full number of rapid tests is reached after the Easter holidays in all scenarios. For rapid tests in the workplace ($\pi_{w,\, s,\, t}$) and private rapid tests ($\pi_{private,\, t}$) it is reached between May 1 and June 10, depending on the scenario.

Moreover, the out of sample predictions assume that the share of detected cases (ψ_t) that would have been obtained without rapid tests is not affected by the Easter holidays because the extent to which this was the case was estimated from case numbers in April.

The results of the out of sample prediction are displayed in Figure B.8. While all scenarios considerably deviate from the ex-post scenario, they all reproduce the steep increase of cases until the end of April, followed by a decline until June. We can therefore conclude that our main results are not sensitive to measurement errors in the number of rapid tests, vaccinations or mobility data.

Another form of validating our model is to see how well our main results align with other studies that evaluate the effect of large scale rapid testing. Of course, this has to be taken with a grain of salt as the effect of any rapid testing policy depends on the incidence of the disease in the population, how well other testing policies such as PCR tests are working, the effect of seasonality and NPIs that are in place.

Nevertheless, it is reassuring that other studies find effect sizes in the same order of magnitude.

Pavelka *et al.* (*108*) estimate that a mass testing campaign in Slovakia in October and November 2020 where approximately 65 % of the population took a rapid test within a two week period lead to a reduction in case numbers of 70 % three weeks after the start of the intervention. Moreover, they find that this strong reduction in cases cannot be explained by isolation of people who tested positive alone but only when they took into account that household members of people who tested positive reduced their contacts.

While we do not model the exact scenario of Pavelka *et al.* (*108*), we can roughly compare their estimates with our predictions for the difference between the baseline

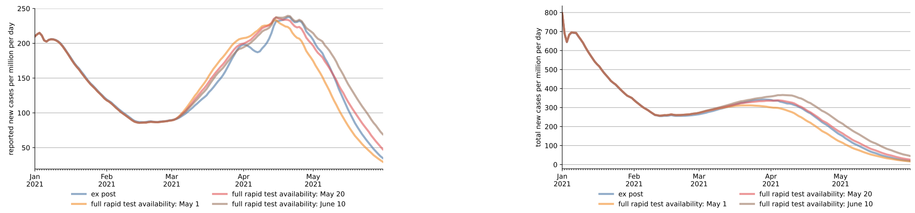

(a) Reported Cases

(b) Total Cases

Figure B.8. Out of Sample Prediction for Reported and Total Cases from March to June 2021.

Note: The ex-post scenario is an in-sample prediction that uses all available information and is very close to actual case numbers. For the other scenarios data on vaccinations, work mobility and rapid tests that became available after March 1 have been replaced by prediction models that are calibrated with data from February. Moreover, they do not model a lower number of detected cases over the Easter holidays. The different scenarios make different assumptions on the date at which full availability of rapid tests is reached. While the out of sample predictions differ substantially for the exact case numbers at the beginning of June (between 20 and 70 cases per million), they can all reproduce the decline in case numbers that is jointly driven by seasonality, large scale rapid tests and vaccinations. For legibility reasons, all lines are rolling 7-day averages. Each line is the average over 30 simulation runs.

scenario and and a scenario without rapid tests. In May about 45% of people do at least one rapid test in every week. Taking into account that there are many repeated testers the number of people who do a test within a two week period is probably slightly less than the 65% from the intervention in Slovakia. On the other hand, we have many people who do more than one rapid test in that time which also leads to the detection of cases. Our model predicts that the observed incidence with tests is approximately 65% lower than without tests after three weeks. Thus we have an effect size in the same order of magnitude but are slightly less optimistic regarding the efficiency of rapid tests.

Berger *et al.* (*109*) analyse the effect of twice weekly rapid testing in schools. They have two main findings: Firstly, rapid tests reduced the share of undetected cases among students by a factor between two and four. Secondly, open schools with mandatory testing might lead to the same or even lower numbers of infections than closed schools. The estimates are based on infection numbers after the Easter holiday.

Again, we do not directly simulate their scenarios but can roughly compare our results to theirs. We estimate a share of undetected cases of approximately 75% among school age children (five to 14 years) at the beginning of April, see Figure B.11. This drops to slightly less than 40% at the end of our simulation period. Thus in the long run, mandatory tests at schools led to a reduction of the share of undetected cases by a factor of more than 1.8 which is just slightly below the factor of two to four predicted by Berger *et al.* (*109*).

Similarly we are slightly less optimistic for the effect of opening schools with testing compared to closing schools. While they predict that opening schools could

even be beneficial we estimate that it would lead to a slight increase in case numbers see Figure B.16).

B.12 Robustness to assumptions about rapid test sensitivity

Our main results are based on rapid test sensitivities read from clinical trials. Recent studies showing that the actual sensitivity of rapid tests may be lower than that (e.g., *110*).

This section shows that our results are robust to making less favorable assumptions on rapid test sensitivity. We proceed by describing several possible ways of calibrating rapid test sensitivity profiles based on recent studies. Since none of these methods is inherently better than the others, we make simulations with two sensitivity profiles: The average over all methods and the lower envelope over all methods using recent studies.

Both profiles imply lower sensitivities of rapid tests than used in our main results. This is especially true during the later stage of an infection. However, the main results stay very robust. The original result was that rapid tests, seasonality and vaccinations are responsible for 42%, 43% and 16%, respectively. With the average profile, the effect of rapid tests decreases to 41 %. With the lower envelope profile, which is an extremely unfavorable assumption, it becomes 38%.

The effect of rapid tests on infection dynamics strongly depends on *when* an infection is detected. Earlier detection means that it is more likely that the infection has not yet been discovered for a different reason (e.g. due to the onset of symptoms) and that the infected person can be isolated before spreading the disease to others.

The sensitivity of rapid tests depends on the viral load in the respiratory tract. It is low at the beginning of an infection (especially before the onset of infectiousness), high in the first few days of infectiousness and then gradually decreasing towards the end of infectiousness.

We thus need to calibrate a profile of rapid test sensitivities based on the number of days until or since the onset of infectiousness.

Unfortunately, such sensitivity profiles are not usually reported in studies. We thus need to create them by combining two types of studies: 1. Studies that report the viral load in terms of threshold cycle (*Ct*) values determined by PCR test (e.g. *111*–*115*). 2. Studies that report the sensitivity of rapid tests for different *Ct* values (e.g. *73*, *110*).

It is natural to assume that the evolution of *Ct* values over time as well as the effect of *Ct* values on rapid test sensitivity are continuous functions. However, the results of the available studies are usually reported in a discretized way. This leads to multiple ways of calculating the sensitivity profiles. Some try to recover the underlying continuous functions using interpolation or regression, others simply use the discretized values.

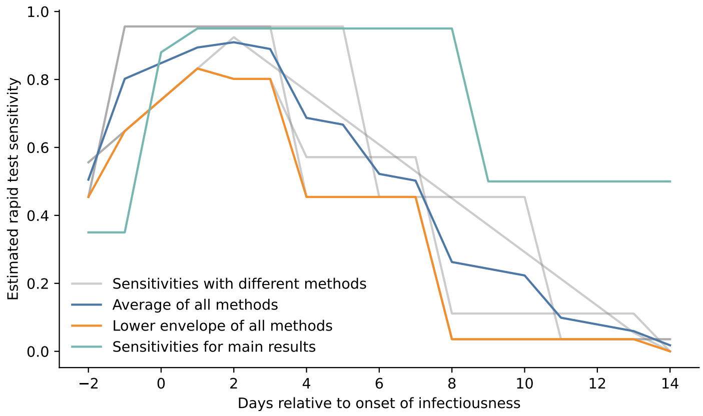

Figure B.9. Rapid test sensitivity profiles

Note: The figure shows estimated sensitivities of rapid tests over the course of an infection. The x-axis shows days relative to the onset of infectiousness. The y-axis shows the estimated rapid test sensitivities. The grey lines are the raw sensitivity estimates obtained with different methods of dealing with discretized data. The blue line shows their average and the yellow line their lower envelope. The turquoise line are the test sensitivities used for the main results of the paper.

For the calibration of *Ct* values over time we can either use discretized values for several time bins from (*112*) and (*114*). Alternatively, we can use linearized formulas for calculating sensitivities over time (*111*) and complement it with interpolations of data points from (*114*) in the pre-infectious stage. Throughout we assume that the *Ct* values of individuals who eventually develop symptoms and those who do not follow the same trajectory. This is in line with results by (*115*) and recent evidence that rapid tests excel at discovering asymptomatic cases (*116*).

For the mapping of *Ct* values to rapid test sensitivities, again we have two options. First, we can simply look up the discretized values for the three *Ct* bins provided in (*110*) (below 20, 25 to 30 and above 35). Secondly, we can use linear regression to estimate a continous mapping for the relationship by assuming that the *Ct* values of each bin are achieved exactly at the bin midpoints and that the relationship is linear.

In general, using discretized values can lead to an underestimation of peak sensitivities and an overestimation of very low sensitivities. This is because discretization is essentially a smoothing device. On the other hand, it has the advantage of simply working with published results, without introducing any tuning parameters or other assumptions.

Figure B.9 shows that the updated sensitivity estimates are lower than the ones used for our original results, especially towards the end of an infection. However, the main results barely change. This is due to the fact that the differences are largest towards the later stage of an infection. Uncovering an infection that was previously undetected at that stage does not have a large effect on infection dynamics.

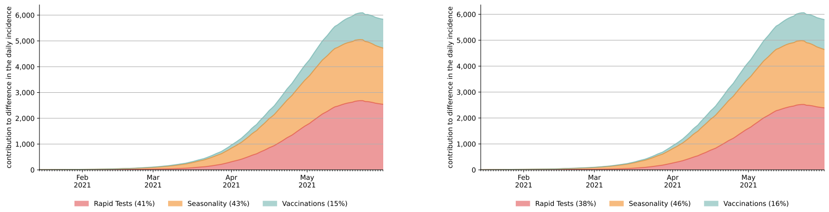

(a) Shapley decomposition with average of sensitivity estimates

(b) Shapley decomposition with lower envelope of sensitivity estimates

Figure B.10. Shapley decompositions for different values of rapid test sensitivity

Note: The figure shows updated versions of the Shapley decomposition in figure 3c. The decompositions are based on 20 model runs with different random seeds. The share attributed to each channel is rounded to the next full percentage point to acknowledge the remaining sampling uncertainty.

B.13 Share of Detected Cases

This section shows the share of detected cases for different age groups. See Section B.7 for an explanation of how we model the detection of cases and Section A.7 for the calibration of the relevant parameters.

The share of detected cases fall drastically from October to December when the incidence of CoViD-19 skyrocketed, PCR tests were still scarce and official contact tracing became impossible due to the sheer amount of cases.

As rapid tests become available and more and more individuals receive positive rapid tests and seek PCR tests, the share of detected cases starts to increase. While first rapid tests are available since the beginning of 2021 the effect only becomes substantial after March when access to rapid tests was greatly expanded.

Overall, the share of detected cases is much higher in older age groups. This is because the likelihood to develop symptoms increases with age and symptomatic cases are more likely to be detected.

A notable exception is that school age children (5-14, green line) overtake the next age group in May 2021. This comes from a particularly strong increase in their share of detected cases after Easter, when weekly rapid tests become mandatory in schools.

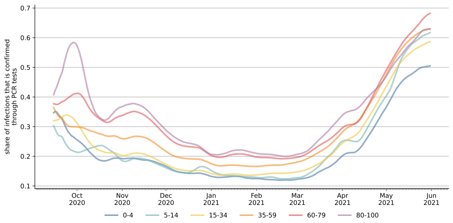

Figure B.11. Share of Detected Cases by Age Group

Note: The figure shows the share of cases that is reported as an official case for each age group in our simulated population. For legibility reasons, all lines are rolling 7-day averages of the average of 30 simulation runs.

B.14 Simulated Rapid Tests

In order to make the most use out of limited data sources on rapid test usage, we model the number of performed rapid tests as a result of time invariant willingness to do rapid tests and time varying supply side factors and events that trigger rapid tests. Thus, the π parameters governing when individuals do rapid tests described in Section A.6 are only indirectly related to the number of rapid tests that are actually performed in the model. When it comes to positive and negative rapid tests, there is even an additional layer because rapid tests are imperfectly sensitive and specific.

In this section we look at how rapid tests expanded in our simulations over time and to what degree they are useful as a screening device despite their imperfections.

We start with the share of the population doing a rapid test and receiving a positive rapid test over time by the channel through which the test was demanded in Figures B.12a, B.12b, respectively. Overall, the share of the population getting a rapid test on a given day increases from 2% in mid March to over 10% by May. The work rapid tests are a little ragged because of public holidays. For education rapid tests both vacations (first half of April) as well as the opening of schools in May are very visible in the rapid test demand. Overall, work tests make up the largest fraction of rapid tests. The image is very similar for the share of positive tests, except that the overall number of positive tests starts decreasing in May as rapid test expansion comes to a halt and cases fall, especially the positive share of private rapid tests falls as less and less individuals are triggered to seek a rapid test because of a risk contact in their household.

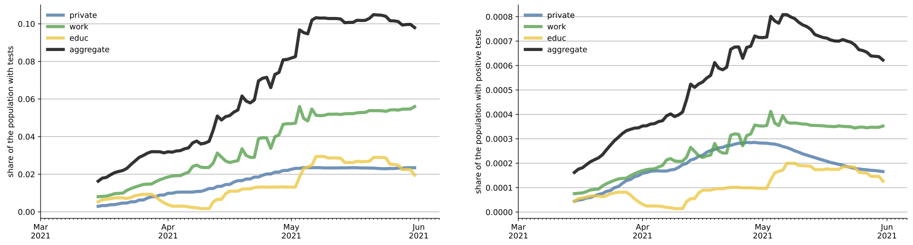

(a) Share of the Population Doing a Rapid Test on a Given Day, by Channels

(b) Share of the Population Testing Positive on a Given Day, by Channels

Figure B.12. Rapid Test Shares in the Population by Channel

Note: Rapid tests in the education setting are demanded by teachers (nursery, preschool and school) as well as pupils. After Easter the required frequency of tests is increased from once per week to twice per week. Work rapid tests are demanded by individuals that still have work contacts, i.e. do not work from home. The share of employers offering rapid tests increases over the time frame and the frequency of testing is also increased. Private tests are demanded by individuals for one of three reasons: having developed symptoms without access to a PCR test, having a household member that has tested positive or developed symptoms or having planned a weekly meeting with friends. Panel a shows the share of the population doing a rapid test on a given day. Panel b shows the share of the population testing positive on a given day (true and false positives).

Next, we show the tests split by whether they are true positive, false positive, true negative or false negative (see Figure B.13) in numbers per million individuals to make the metric comparable to incidences.

The number of true positives (Figure B.13a) rapidly increases and peaks at the end of April with over 200 cases per million detected through rapid tests per day. This means that our model suggests that Germany was able to detect up to 16,600 cases per day that would have likely gone undetected otherwise. The most powerful tool for detecting cases are the private rapid tests. This is because a large share of them are targeted, i.e. triggered by events in the household. However, this does not mean that rapid tests in the workplace or at school are less important. It is rather the combination of large scale screening at work and in schools and very efficient follow up tests whenever those screening tests detected a case. Shapley values (Figure 3d) take this into account and assign about 50% of the overall reduction of case numbers via rapid tests to private rapid tests with work and school rapid tests accounting for 40% and 7%, respectively.

Such a large effect of rapid tests seems to be at odds with the general perception that they are not very reliable. However, one has to differentiate between the reliability of one test in isolation and the effect imperfect tests can have when employed at a large scale. On average our tests have a sensitivity of slightly more than 70%. This means they miss almost 30% of infections among the tested. Of course perfect tests would have an even larger effect but the relevant number to compare is that up to 200 cases per million are detected by rapid tests every day which would have otherwise gone undetected.

This clearly shows that the large effect of rapid tests on the infection dynamic is not driven by unrealistic assumptions about their sensitivity but rather by the fact that there was a very large number of infected individuals who did not know they are infected. Detecting and isolating some of them is enough to slow down the overall infection dynamic.

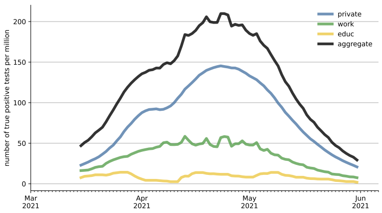

Negative **(a)** Number of Discovered Cases Due to Rapid Tests by Channel

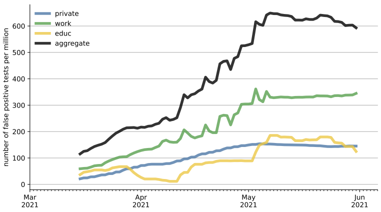

(b) Number of False Positive Rapid Tests by Channel

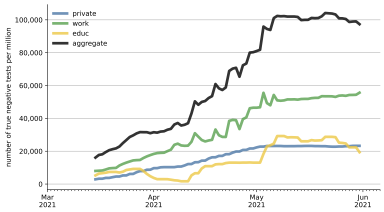

(c) Number of True Negative Rapid Tests by Channel

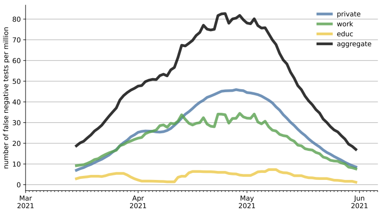

(d) Number of False Negative Rapid Tests by Channel

Figure B.13. Simulated Rapid Test Statistics

Note: Each panel shows the number of rapid tests per million inhabitants that fall into the respective category. Private rapid tests are especially good at detecting cases but since they are often triggered by rapid tests from other channels, the other groups of tests, especially rapid tests at the workplace, also play an important role for containing the pandemic. All results are averaged over 30 simulation runs. For legibility reasons, all lines are rolling 7-day averages.

A similar picture arises, when looking at the false positive rate, i.e. the share of positive tests that go to people who are not infected. Figure B.14a shows that the false positive rate is very high. On average 60% to 93% of positive tests are received by individuals that are not infected. The false positive rate increases over time. This is due to the low prevalence of infections in the population, which falls over time. Again, private rapid tests are an exception with a much lower false positive rate because those tests are primarily demanded when there is a high likelihood of being infected. The false negative rate of 0.2% looks very low. As discussed above this is deceiving and just a mechanical consequence of a very low prevalence of the disease and the many rapid tests done by non-infected people.

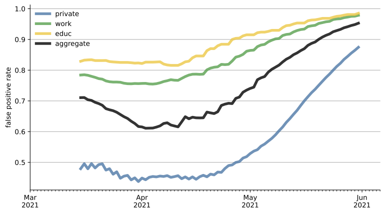

(a) Rate of False Positive Rapid Tests by Channel

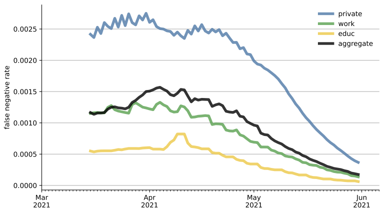

(b) Rate of False Negative Rapid Tests by Channel

Figure B.14. False Positive and False Negative Rates by Channel

Note: The left panel shows the share of positive tests that are given to people who are not infected. This share is large as can be expected with a very low baseline rate of positive individuals. As the incidence in the population drops, the false positive rate increases. An exception are the private rapid tests because they are – especially when the incidence is high – often triggered by events that make it likely that the test taker is infected and therefore their false positive rate is much lower. The right panel shows the false negative rate in the population, i.e. the share of negative tests done by infected individuals. This is very low because there are many truly negative tests in times of low incidences and large scale screening tests.

B.15 Scenarios

Here we complement our analysis of the effectiveness of vaccinations and rapid tests by showing the effects of rapid test policies vis-à-vis the more traditional NPIs, work from home mandates and school closures. All scenarios start after Easter (April 6). Our analyses show that many socially costly NPIs can be avoided through strong rapid testing policies.

Figure B.15 shows the effects of different work policies on the infections in the general population. We compare four scenarios with our baseline scenario: Keeping the share of workers having physical work contacts the same as in our baseline scenario the orange line shows what would have happened with rapid testing in firms at the level of mid March (orange line) where only 14% of workers regularly did rapid tests. We also include a scenario what would have happened if rapid tests had become truly mandatory after Easter[14], assuming a 95% compliance rate on both the employer and the employee side. On the work from home dimension we compare our baseline scenario with 10% more or less work from home compared to the baseline scenario. For the total cases, the picture is very clear. Given the testing policy Germany had in place during that time (twice weekly tests done by 35% to 50% of workers over that time frame) whether 70% (10% below the actual mobility) or 85% (10% above the actual mobility) of workers attend work physically makes little difference for the incidence. On the other hand, the effect of a laxer or more ambitious testing policy for firms is sizable: As can be seen in Figure B.15b the gap between the two scenarios grows to over 80 incidence points around May 1.As in other scenarios, the observed cases can be misleading because more testing leads to more detected cases. It takes two to three weeks for the reduction in new infections to dominate the increased detection. Furthermore, the two opposing effects lead to a smaller effect size than is actually the case.

14. Starting on April 19th employers were required by law to provide two weekly tests to their employees (*53*). However, voluntarily only 60% of workers regularly test themselves when offered tests ((*48*), 20th/21st of April).

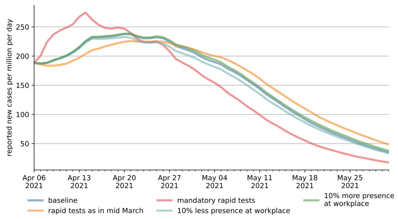

(a) Reported Cases

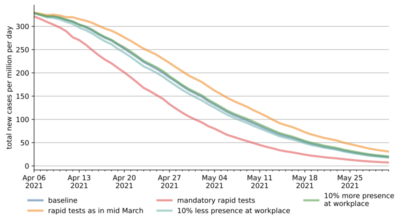

(b) Total Cases

Figure B.15. The Effect of Different Work Scenarios on Reported and Total Cases

Note: The figure shows the development of cases after different hypothetical work policy changes take place at Easter until the end of our simulation period. We vary the share of workers that have physical work contacts (10% more or less compared to the share in the baseline scenario, 85% or 70% of workers, respectively) and how many tests are performed at work relative to our baseline scenario. As an ambitious scenario we implement mandatory tests for all employees that do not work from home, assuming 95% compliance on both the employer and the employee side. On the other hand, we show what would have happened if the test offers had fallen back to the level of mid March (only 14% of workers are tested regularly). The observed cases can be misleading because more testing leads to more detected cases. It takes two to three weeks for the reduction in new infections to dominate the increased detection. Furthermore, the two opposing effects lead to a smaller effect size than is actually the case.

The second commonly employed and also very contentious NPI we look at are school closures. Due to the very high incidence we model the German schooling policy as generous emergency care with rotating on-site schooling for graduating classes for April. In May where cases fall and schools gradually opened, we model the policy as rotating on-site schooling for most students (except for children eligible for emergency care and graduating classes who attend in full). We compare this baseline scenario to simply keeping schools completely closed (the brown line) and opening schools normally (but maintaining our hygiene multiplier to account for mask wearing, ventilation etc.) with and without tests.

As can be seen, the transmission potential in schools is very low both in the generous emergency setting as well as the rotating operation. The difference to keeping schools completely closed is very small. Also, consistent testing reduces the transmission potential at schools strongly. Had schools opened directly after Easter given the testing rates Germany managed at schools during that time, the total incidence would have been only been 9 incidence points higher on average. Tests, however, are crucial here. Had schools opened completely without any testing of students and staff, schools would have added up to 50 incidence points.

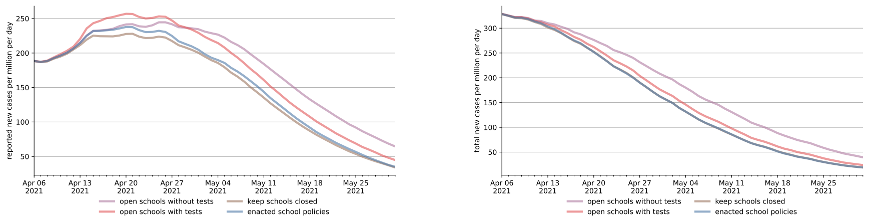

(a) Reported Cases

(b) Total Cases

Figure B.16. The Effect of Different School Scenarios on Reported and Total Cases

Note: The figure shows the development of cases after different hypothetical school policy changes take place at Easter until the end of our simulation period. Apart from the enacted school policies as our baseline we simulate how cases would have developed if schools had been closed completely as the strictest possible counterfactual scenario and two opening models: One where schools open normally (with hygiene measures) without any testing in the education sector and one where schools open normally but testing develops as in the baseline scenario.

Lastly, we shed some light on the role our rapid test demand channels play for the effect of rapid tests on case numbers. To do so we ran two scenarios where we allocated rapid tests either completely randomly in the entire population or among 70% of the population to account for the fact that a share of the population might refuse or be very hard to reach with rapid tests.[15]

Figure B.17 shows how the incidence of detected and total cases develops in the two random scenarios (red and purple line) relative to our baseline scenario (blue line). Two things stand out: Firstly, the total number of cases falls much faster in our baseline scenario compared to the two random scenarios. Secondly, this is not because the share of detected cases is higher in the baseline scenario; in fact, it is even slightly lower until end of April.

There are two mechanisms that can explain these surprising facts: Firstly, tests at the workplace predominantly target a group that has many contacts. Thus, catching infections in this group prevents more infections than in the general population. Secondly, rapid tests that are done because of private contact tracing are more effective at interrupting infection chains because they catch many infections in an early stage. Isolating infected individuals early on means that there are fewer days on which they can infect others. The difference between the two random scenarios are small. This is likely due to only a small fraction of the population being tested on any given day.

15. We calculate the number of rapid tests as in our baseline model. This leads to similar numbers of rapid tests. However given the higher incidence in our random scenarios these scenarios have a slightly higher number of rapid tests.

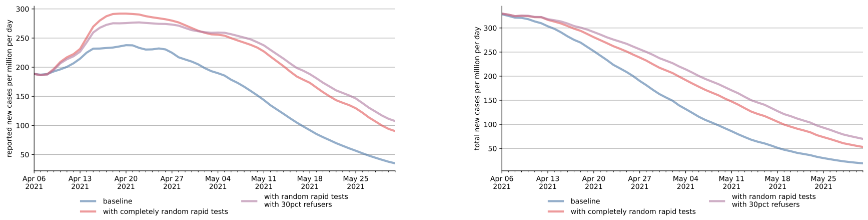

(a) Reported Cases

(b) Total Cases

Figure B.17. The Role of Targeted and Compliance Driven Rapid Test Demand

Note: The figure shows the development of cases in two scenarios where rapid tests are distributed randomly in the population compared to our baseline scenario after Easter. In the baseline scenario rapid tests are targeted to workers, students, teachers and individuals at high risk of being infected including a weekly or twice weekly spacing between rapid tests. In the scenario with 30% refusers tests are randomly distributed among 70% of the population who are identified as compliers.

References

1. N. G. Davies, P. Klepac, Y. Liu, K. Prem, M. Jit, R. M. Eggo, Age-dependent effects in the transmission and control of COVID-19 epidemics. *Nature Medicine* **26**, 1205–1211 (2020).
2. N. G. Davies, S. Abbott, R. C. Barnard, C. I. Jarvis, A. J. Kucharski, J. D. Munday, C. A. B. Pearson, T. W. Russell, D. C. Tully, A. D. Washburne, T. Wenseleers, A. Gimma, W. Waites, K. L. M. Wong, K. van Zandvoort, J. D. Silverman, K. Diaz-Ordaz, R. Keogh, R. M. Eggo, S. Funk, M. Jit, K. E. Atkins, W. J. Edmunds, Estimated transmissibility and impact of SARS-CoV-2 lineage B.1.1.7 in England. *Science* (2021).
3. S. Zhao, B. Tang, S. S. Musa, S. Ma, J. Zhang, M. Zeng, Q. Yun, W. Guo, Y. Zheng, Z. Yang, Z. Peng, M. K. Chong, M. Javanbakht, D. He, M. H. Wang, Estimating the generation interval and inferring the latent period of COVID-19 from the contact tracing data. *Epidemics* **36**, 100482 (2021).
4. C. M. Peak, R. Kahn, Y. H. Grad, L. M. Childs, R. Li, M. Lipsitch, C. O. Buckee, Individual quarantine versus active monitoring of contacts for the mitigation of COVID-19: a modelling study. *The Lancet Infectious Diseases* **20**, 1025–1033, (https://doi.org/10.1016/s1473-3099(20)30361-3) (2020).
5. X. He, E. H. Lau, P. Wu, X. Deng, J. Wang, X. Hao, Y. C. Lau, J. Y. Wong, Y. Guan, X. Tan, *et al.*, Temporal dynamics in viral shedding and transmissibility of COVID-19. *Nature medicine* **26**, 672–675 (2020).
6. C. McAloon, Á. Collins, K. Hunt, A. Barber, A. W. Byrne, F. Butler, M. Casey, J. Griffin, E. Lane, D. McEvoy, P. Wall, M. Green, L. O'Grady, S. J. More, Incubation period of COVID-19: a rapid systematic review and meta-analysis of observational research. *BMJ Open* **10**, e039652, (https://doi.org/10.1136/bmjopen-2020-039652) (2020).

7. G. Yin, H. Jin, Comparison of Transmissibility of Coronavirus Between Symptomatic and Asymptomatic Patients: Reanalysis of the Ningbo COVID-19 Data. *JMIR Public Health and Surveillance* **6**, e19464 (2020).

8. L. Zou, F. Ruan, M. Huang, L. Liang, H. Huang, Z. Hong, J. Yu, M. Kang, Y. Song, J. Xia, Q. Guo, T. Song, J. He, H.-L. Yen, M. Peiris, J. Wu, SARS-CoV-2 Viral Load in Upper Respiratory Specimens of Infected Patients. *New England Journal of Medicine* **382**, 1177–1179 (2020).

9. A. W. Byrne, D. McEvoy, A. B. Collins, K. Hunt, M. Casey, A. Barber, F. Butler, J. Griffin, E. A. Lane, C. McAloon, K. O'Brien, P. Wall, K. A. Walsh, S. J. More, Inferred duration of infectious period of SARS-CoV-2: rapid scoping review and analysis of available evidence for asymptomatic and symptomatic COVID-19 cases. *BMJ Open* **10**, e039856 (2020).

10. A. Singanayagam, M. Patel, A. Charlett, J. L. Bernal, V. Saliba, J. Ellis, S. Ladhani, M. Zambon, R. Gopal, Duration of infectiousness and correlation with RT-PCR cycle threshold values in cases of COVID-19, England, January to May 2020. *Eurosurveillance* **25** (2020).

11. Q. Bi, Y. Wu, S. Mei, C. Ye, X. Zou, Z. Zhang, X. Liu, L. Wei, S. A. Truelove, T. Zhang, W. Gao, C. Cheng, X. Tang, X. Wu, Y. Wu, B. Sun, S. Huang, Y. Sun, J. Zhang, T. Ma, J. Lessler, T. Feng, Epidemiology and transmission of COVID-19 in 391 cases and 1286 of their close contacts in Shenzhen, China: a retrospective cohort study. *The Lancet Infectious Diseases* **20**, 911–919 (2020).

12. E. K. Stokes, L. D. Zambrano, K. N. Anderson, E. P. Marder, K. M. Raz, S. E. B. Felix, Y. Tie, K. E. Fullerton, Coronavirus Disease 2019 Case Surveillance — United States, January 22–May 30, 2020. *MMWR. Morbidity and Mortality Weekly Report* **69**, 759–765 (2020).

13. R. Hinch, W. J. M. Probert, A. Nurtay, M. Kendall, C. Wymatt, M. Hall, K. Lythgoe, A. B. Cruz, L. Zhao, A. Stewart, L. Ferritti, D. Montero, J. Warren, N. Mather, M. Abueg, N. Wu, A. Finkelstein, D. G. Bonsall, L. Abeler-Dorner, C. Fraser, *OpenABM-Covid19 - Parameter Dictionary* (https://github.com/BDI-pathogens/OpenABM-Covid19/blob/14e24ce298ad523eba5e6f33ee9c70a90c527b3e/documentation/parameters/parameter_dictionary.md).

14. J. Chen, T. Qi, L. Liu, Y. Ling, Z. Qian, T. Li, F. Li, Q. Xu, Y. Zhang, S. Xu, Z. Song, Y. Zeng, Y. Shen, Y. Shi, T. Zhu, H. Lu, Clinical progression of patients with COVID-19 in Shanghai, China. *Journal of Infection* **80**, e1–e6 (2020).

15. K. Gaythorpe, N. Imai, G. Cuomo-Dannenburg, M. Baguelin, S. Bhatia, A. Boonyasiri, A. Cori, Z. Cucunuba Perez, A. Dighe, I. Dorigatti, R. Fitzjohn, H. Fu, W. Green, J. Griffin, A. Hamlet, W. Hinsley, N. Hong, M. Kwun, D. Laydon, G. Nedjati Gilani, L. Okell, S. Riley, H. Thompson, S. Van Elsland, R. Verity, E. Volz, P. Walker, H. Wang, Y. Wang, C. Walters, C. Whittaker, P. Winskill, X. Xi, C. Donnelly, A. Ghani, N. Ferguson, Report 8: Symptom progression of COVID-19. (2020).

16. L. J. Abu-Raddad, H. Chemaitelly, J. A. Malek, A. A. Ahmed, Y. A. Mohamoud, S. Younuskunju, H. H. Ayoub, Z. A. Kanaani, A. A. Khal, E. A. Kuwari, A. A. Butt, P. Coyle, A. Jeremijenko, A. H. Kaleeckal, A. N. Latif, R. M. Shaik, H. F. A. Rahim, H. M. Yassine, M. G. A. Kuwari, H. E. A. Romaihi, M. H. Al-Thani, R. Bertollini, Assessment of the Risk of Severe Acute Respiratory Syndrome Coronavirus 2 (SARS-CoV-2) Reinfection in an Intense Reexposure Setting. *Clinical Infectious Diseases* (2020).

17. impfdashboard.de, *Download der Datensätze* (https://impfdashboard.de/daten).

18. E. Pritchard, P. C. Matthews, N. Stoesser, D. W. Eyre, O. Gethings, K.-D. Vihta, J. Jones, T. House, H. VanSteenHouse, I. Bell, J. I. Bell, J. N. Newton, J. Farrar, I. Diamond, E. Rourke, R. Studley, D. Crook, T. E. A. Peto, A. S. Walker, K. B. Pouwels, Impact of vaccination on new SARS-CoV-2 infections in the United Kingdom. *Nature Medicine* (2021).

19. R. Harris, J. Hall, A. Zaidi, N. Andrews, J. K. Dunbar, G. Dabrera, "Impact of vaccination on household transmission of SARS-COV-2 in England," tech. rep. (Public Health England, 2021), (https://khub.net/documents/135939561/390853656/Impact%20of%20vaccination%20on%20household%20transmission%20of%20SARS-COV-2%20in%20England.pdf/35bf4bb1-6ade-d3eb-a39e-9c9b25a8122a?t=1619601878136).

20. E. Petter, O. Mor, N. Zuckerman, D. Oz-Levi, A. Younger, D. Aran, Y. Erlich, Initial real world evidence for lower viral load of individuals who have been vaccinated by BNT162b2. *medRxiv* (2021).

21. M. Levine-Tiefenbrun, I. Yelin, R. Katz, E. Herzel, Z. Golan, L. Schreiber, T. Wolf, V. Nadler, A. Ben-Tov, J. Kuint, S. Gazit, T. Patalon, G. Chodick, R. Kishony, Initial report of decreased SARS-CoV-2 viral load after inoculation with the BNT162b2 vaccine. *Nature medicine* **27**, 790–792 (2021).

22. P. R. Hunter, J. Brainard, Estimating the effectiveness of the Pfizer COVID-19 BNT162b2 vaccine after a single dose. A reanalysis of a study of real-world vaccination outcomes from Israel. *medRxiv* (2021).

23. M. G. Thompson, J. L. Burgess, A. L. Naleway, H. L. Tyner, S. K. Yoon, J. Meece, L. E. Olsho, A. J. Caban-Martinez, A. Fowlkes, K. Lutrick, *et al.*, Interim estimates of vaccine effectiveness of BNT162b2 and mRNA-1273 COVID-19 vaccines in preventing SARS-CoV-2 infection among health care personnel, first responders, and other essential and frontline workers—eight US locations, December 2020–March 2021. *Morbidity and Mortality Weekly Report* **70**, 495, (https://www.cdc.gov/mmwr/volumes/70/wr/mm7013e3.htm?s_cid=mm7013e3_w) (2021).

24. M. Lipsitch, R. Kahn, Interpreting vaccine efficacy trial results for infection and transmission. *Vaccine* **39**, 4082–4088, ISSN: 0264-410X, (https://www.sciencedirect.com/science/article/pii/S0264410X21007350) (2021).

25. Forschungsdatenzentren Der Statistischen Ämter Des Bundes Und Der Länder, *Mikrozensus 2010, CF, Version 0*, de, 2018.

26. Destatis, "Betreuungsquote von Kindern unter 6 Jahren nach Bundesländern," tech. rep. (Statistisches Bundesamt, 2020), (https://www.destatis.de/DE/Themen/Gesellschaft-Umwelt/Soziales/Kindertagesbetreuung/Tabellen/betreuungsquote-2018.html).

27. Bertelsmann Stiftung, "Ländermonitor Frühkindliche Bildungssysteme 2019," tech. rep. (Bertelsmann Stiftung, 2019), (https://www.laendermonitor.de/fileadmin/files/Projekte/Laendermonitoring_Fruehkindliche_Bildungssysteme/Laendermonitor_2019/IN_Keyfacts_alle_Bundeslaender_2019_01.pdf).

28. OECD, *Bildung auf einen Blick 2013: OECD-Indikatoren*, p. 530, (https://www.oecd-ilibrary.org/content/publication/eag-2013-de).

29. J. Mossong, N. Hens, M. Jit, P. Beutels, K. Auranen, R. Mikolajczyk, M. Massari, S. Salmaso, G. S. Tomba, J. Wallinga, *et al.*, Social contacts and mixing patterns relevant to the spread of infectious diseases. *PLoS medicine* **5** (2008).

30. Robert Koch-Institut, "COVID-19 Impfquoten-Monitoring in Deutschland (COVIMO): Report 5 - Fokuserhebung Impfquoten," tech. rep., (https://www.rki.de/DE/Content/InfAZ/N/Neuartiges_Coronavirus/Projekte_RKI/COVIMO_Reports/covimo_studie_bericht_5.pdf?__blob=publicationFile).

31. S. Vygen-Bonnet, J. Koch, C. Bogdan, T. Harder, U. Heininger, K. Kling, M. Littmann, J. Meerpohl, H. Meyer, T. Mertens, N. Schmid-Küpke, S. Scholz, M. Terhardt, M. Treskova-Schwarzbach, K. Überla, M. van der Sande, O. Wichmann, S. Wicker, U. Wiedermann, V. Wild, R. von Kries, Beschluss und Wissenschaftliche Begründung der Ständigen Impfkommission (STIKO) für die COVID-19-Impfempfehlung. 3–63 (2020).

32. Bundesgesetzblatt, *Viertes Gesetz zum Schutz der Bevölkerung bei einer epidemischen Lage von nationaler Tragweite vom 22. April 2021*, 2021, (https://www.bundesgesundheitsministerium.de/fileadmin/Dateien/3_Downloads/Gesetze_und_Verordnungen/GuV/B/4_BevSchG_BGBL.pdf).

33. Landesregierung von Baden-Württemberg, *Coronavirus: Aktuelle Informationen für Schulen und Kindertageseinrichtungen*, Accessed 2021-03-25. (2021; https://km-bw.de/Coronavirus).

34. Landesregierung von Baden-Württemberg, *Präsenzbetrieb und inzidenzunabhängige Testpflicht ab dem 19. April* (2021; https://km-bw.de/,Lde/startseite/service/2021-04-15-schulbetrieb-ab-dem-19april).

35. Landesregierung von Baden-Württemberg, *Auswirkungen der Bundesnotbremse auf den Schul- und Kitabetrieb in Baden-Württemberg* (https://km-bw.de/Lde/startseite/service/2021-04-23+Auswirkungen+der+Bundesnotbremse+auf+den+Schul-+und+Kitabetrieb+in+Baden-Wuerttemberg).

36. Bayerisches Staatsministerium für Unterricht und Kultus, *Informationen zum Unterrichtsbetrieb nach den Osterferien* (https://www.km.bayern.de/lehrer/meldung/7244/informationen-zum-unterrichtsbetrieb-nach-den-osterferien.html).

37. Bayerisches Staatsministerium für Unterricht und Kultus, *FAQ zum Unterrichtsbetrieb an Bayerns Schulen*, Accessed on 2021-04-30 (https://www.km.bayern.de/allgemein/meldung/7047/faq-zum-unterrichtsbetrieb-an-bayerns-schulen.html).
38. Ministerium für Schule und Bildung des Landes Nordrhein-Westfalen, *[08.04.2021] Informationen zum Schulbetrieb in NRW* (2021; https://schulministerium.nrw/08042021-informationen-zum-schulbetrieb-nrw).
39. Ministerium für Schule und Bildung des Landes Nordrhein-Westfalen, *[22.04.2021] Informationen zum Schulbetrieb ab 26. April 2021* (2021; https://www.schulministerium.nrw/22042021-informationen-zum-schulbetrieb-ab-26-april-2021).
40. Ministerium für Schule und Bildung des Landes Nordrhein-Westfalen, *„Notbremse" im Infektionsschutzgesetz des Bundes* (2021; https://schulministerium.nrw/presse/pressemitteilungen/notbremse-im-infektionsschutzgesetz-des-bundes-23-04-2021).
41. Ministerium für Schule und Bildung des Landes Nordrhein-Westfalen, *Pressemitteilung: Ministerin Gebauer: Bleiben auf dem Weg der Vorsicht: Wechselunterricht mit klaren Testvorgaben ab Montag* (2021; https://schulministerium.nrw/presse/pressemitteilungen/ministerin-gebauer-bleiben-auf-dem-weg-der-vorsicht-14-04-2021).
42. Bayerisches Staatsiministerium für Familie, Arbeit und Soziales, *Kindertagesbetreuung: Aktuelle Informationen zum Coronavirus (SARS-CoV-2)* (2021; https://www.stmas.bayern.de/coronavirus-info/corona-kindertagesbetreuung.php).
43. Ministerium für Kinder, Familie, Flüchtlinge und Integration des Landes Nordrhein-Westfalen, *Verordnung zum Schutz vor Neuinfizierungen mit dem Coronavirus SARS-CoV-2im Bereich der Betreuungsinfrastruktur (Coronabetreuungsverordnung – CoronaBetrVO)Vom 7. Januar 2021: In der ab dem 8. März 2021 gültigen Fassung*, (https://www.mkffi.nrw/sites/default/files/asset/document/2021-03-07_coronabetrvo_ab_08.03.2021_lesefassung.pdf).
44. Landesregierung von Baden-Württemberg, *Pressemitteilung: Landesweite Schließung von Schulen und Kindergärten* (2021; https://www.baden-wuerttemberg.de/de/service/presse/pressemitteilung/pid/landesweite-schliessung-von-schulen-und-kindergaerten/).
45. Landesregierung von Baden-Württemberg, *Pressemitteilung: Schnelltests in Kitas und Kindergärten* (2021; https://www.baden-wuerttemberg.de/de/service/presse/pressemitteilung/pid/schnelltests-in-kitas-und-kindergaerten/).
46. Bayerisches Staatsiministerium für Familie, Arbeit und Soziales, *404. Newsletter: Allgemeine Informationen zur Kindertagesbetreuung: Informationen zum Coronavirus (SARS-CoV-2)* (2021; https://www.stmas.bayern.de/imperia/md/content/stmas/stmas_inet/service-kinder/newsletter/404-newsletter.pdf).

47. Google, LLC, “Google COVID-19 Community Mobility Reports,” tech. rep., (https://www.google.com/covid19/mobility/).

48. C. Betsch, L. Korn, L. Felgendreff, S. Eitze, P. Schmid, P. Sprengholz, L. Wieler, P. Schmich, V. Stollorz, M. Ramharter, M. Bosnjak, S. B. Omer, H. Thaiss, F. De Bock, U. Von Rüden, *COVID-19 Snapshot Monitoring (COSMO Germany) - Wave 41*, 2021.

49. Deutscher Industrie- und Handelskammertag, “Umfrage der IHK-Organisation zu Corona-Tests in den Unternehmen,” tech. rep., (https://www.dihk.de/resource/blob/48068/e4fd68d36b074e63530cbe34f98e318a/startumfrage-coronatests-data.pdf).

50. E. Ahlers, M. Lübker, R. Jung, “Schleppender Start für wöchentliche Corona-Schnelltests am Arbeitsplatz: Nur 23 Prozent der Beschäftigten haben schon Zugang,” tech. rep. (Wirtschafts- und Sozialwissenschaftliches Institut, 2021), (https://www.boeckler.de/de/pressemitteilungen-2675-schleppender-start-fur-wochentliche-corona-schnelltests-am-arbeitsplatz-31982.htm).

51. Bundesministerium für Wirtschaft und Energie, *Corona-Tests in der Wirtschaft: Ergebnisse der Umfragen bei Beschäftigten und Unternehmen*, Pressemitteilung (2021; https://www.bmwi.de/Redaktion/DE/Pressemitteilungen/2021/04/20210408-corona-tests-in-der-wirtschaft-ergebnisse-umfragen.html).

52. H. Bonin, A. Krause-Pilatus, U. Rinne, “Verbreitung von Homeoffice und Corona-Tests der Arbeitgeber Ende März-Anfang April 2021, Ergebnisse einer repräsentativen Befragung von abhängig Beschäftigten,” Forschungsbericht 570/3, (2021; https://www.bmas.de/SharedDocs/Downloads/DE/Publikationen/Forschungsberichte/fb-570-3-verbreitung-homeoffice-corona-tests-ende-maerz-april-2021.pdf;jsessionid=C652579EFEAB79C3DA916404FA8AAC75.delivery1-replication?__blob=publicationFile&v=3).

53. Bundesanzeiger, *Zweite Verordnung zur Änderung der SARS-CoV-2-Arbeitsschutzverordnung*, 2021, (https://www.bmas.de/SharedDocs/Downloads/DE/Gesetze/zweite-aenderungsverordnung-sars-cov-2-arbeitsschutzverordnung.pdf).

54. Bayerisches Staatsministerium für Gesundheit und Pflege, *Holetschek: Bayern hat frühzeitig Selbsttests gesichert - Schulen und Kitas bekommen schon nächste Woche 1,3 Millionen Selbsttests*, Pressemitteilung Nr. 44/GP (2021; https://www.stmgp.bayern.de/presse/holetschek-bayern-hat-fruehzeitig-selbsttests-gesichert-schulen-und-kitas-bekommen-schon/).

55. Ministerium für Kultus, Jugend und Sport Baden Württemberg, Erweiterte Teststrategie - Selbsttests an Schulen. *Pressemitteilungen*, (https://km-bw.de/,Lde/startseite/service/2021-04-08-selbsttests-an-schulen) (2021).

56. Ministerium für Schule und Bildung des Landes Nordrhein-Westfalen, *Umfassende Informationen für die Schulen zu Corona-Selbsttests für Schülerinnen und Schüler*, Pressemitteilung (2021; https://www.land.nrw/de/pressemitteilung/umfassende-informationen-fuer-die-schulen-zu-corona-selbsttests-fuer-schuelerinnen).

57. Niedersächsisches Kultusministerium, *Selbsttests an Schulen laufen an - Auslieferung der ersten 400.000 Tests beginnt heute, Testwoche startet Montag*, Pressemitteilung (2021; https://www.mk.niedersachsen.de/startseite/aktuelles/presseinformationen/selbsttests-an-schulen-laufen-an-erste-400-000-tests-ab-heute-in-der-auslieferung-testwoche-startet-montag-198555.html).

58. Sächsisches Staatsministerium für Kultus, *Selbsttests für Schüler und Lehrer ab Mittwoch* (2021; https://www.medienservice.sachsen.de/medien/news/248791).

59. Bayerische Staatskanzlei, *Bericht aus der Kabinettssitzung vom 7. April 2021*, Pressemitteilung (2021; https://www.bayern.de/bericht-aus-der-kabinettssitzung-vom-7-april-2021/?seite=2453).

60. Ministerium für Schule und Bildung des Landes Nordrhein-Westfalen, *Schulbetrieb im Wechselunterricht ab Montag, 19.April 2021 // Coronaselbsttests an Schulen - Testpflicht* (2021; https://www.schulministerium.nrw/14042021-schulbetrieb-im-wechselunterricht-ab-montag-19-april-2021-coronaselbsttests-schulen).

61. Ministerium für Kultus, Jugend und Sport Baden-Württemberg, *Informationen zum Schulbetrieb nach den Osterferien*, Pressemitteilung (2021; https://km-bw.de/,Lde/9112691).

62. Bundesanzeiger, *Verordnung zum Anspruch auf Testung in Bezug auf einen direkten Erregernachweis des Coronavirus SARS-CoV-2 (Coronavirus-Testverordnung - TestV)*, 2021, (https://www.bundesgesundheitsministerium.de/fileadmin/Dateien/3_Downloads/C/Coronavirus/Verordnungen/Corona-TestV_BAnz_AT_09.03.2021_V1.pdf).

63. Presse- und Informationsamt der Bundesregierung, *Erste Zulassungen für Selbsttests* (2021; https://www.bundesregierung.de/breg-de/themen/coronavirus/zulassung-schnell-test-1861354).

64. C. Betsch, L. Korn, L. Felgendreff, S. Eitze, P. Schmid, P. Sprengholz, L. Wieler, P. Schmich, V. Stollorz, M. Ramharter, M. Bosnjak, S. B. Omer, H. Thaiss, F. De Bock, U. von Rüden, *Zusammenfassung und Empfehlungen Welle 37* (2021; https://projekte.uni-erfurt.de/cosmo2020/web/summary/37/).

65. C. Paul, A. Steinberg, C. Schaller, K. Haemmerl, L. Thum, M. Parciak, M. Knaf, S. Meyer, T. Parciak, U. Grupp, *Dunkelzifferradar*, ed. by P. Fund (https://prototypefund.de/project/dunkelzifferradar/).

66. Robert Koch Institute, *Testkriterien für die SARS-CoV-2 Diagnostik: Anpassungen für die Herbst- und Wintersaison 2020/2021* (https://web.archive.org/web/20201103122248/https://www.rki.de/DE/Content/InfAZ/N/Neuartiges_Coronavirus/Teststrategie/Testkiterien_Herbst_Winter.html).
67. Robert Koch Instititue, *COVID-19-Fälle nach Meldewoche und Geschlecht sowie Anteile mit für COVID-19 relevanten Symptomen, Anteile Hospitalisierter/Verstorbener und Altersmittelwert/-median* (https://www.rki.de/DE/Content/InfAZ/N/Neuartiges_Coronavirus/Daten/Klinische_Aspekte.xlsx).
68. Robert Koch-Institut, *Laborbasierte Surveillance SARS-CoV-2* (https://ars.rki.de/Content/COVID19/Main.aspx).
69. D. McFadden, A method of simulated moments for estimation of discrete response models without numerical integration. *Econometrica: Journal of the Econometric Society*, 995–1026 (1989).
70. L. S. Shapley, *17. A value for n-person games* (Princeton University Press, 2016).
71. T. Gavenčiak, J. T. Monrad, G. Leech, M. Sharma, S. Mindermann, J. M. Brauner, S. Bhatt, J. Kulveit, Seasonal variation in SARS-CoV-2 transmission in temperate climates. *medRxiv*, (https://www.medrxiv.org/content/early/2021/06/13/2021.06.10.21258647) (2021).
72. J. Frisch, *Deutsche mit hoher Corona-Impfbereitschaft – aber Wissenslücken* (https://www.aerztezeitung.de/Politik/Deutsche-mit-hoher-Corona-Impfbereitschaft-aber-Wissensluecken-417569.html).
73. L. E. Brümmer, S. Katzenschlager, M. Gaeddert, C. Erdmann, S. Schmitz, M. Bota, M. Grilli, J. Larmann, M. A. Weigand, N. R. Pollock, *et al.*, Accuracy of novel antigen rapid diagnostics for SARS-CoV-2: A living systematic review and meta-analysis. *PLoS medicine* **18**, e1003735 (2021).
74. Anaconda, *Anaconda Software Distribution*, version Vers. 2-2.4.0, 2016, (https://docs.anaconda.com/).
75. conda-forge community, *The conda-forge Project: Community-based Software Distribution Built on the conda Package Format and Ecosystem*, 2015, (https://doi.org/10.5281/zenodo.4774216).
76. M. Rocklin, presented at the Proceedings of the 14th python in science conference, vol. 130, p. 136.
77. J. Gabler, "A Python Tool for the Estimation of (Structural) Econometric Models.," 2020, (https://github.com/OpenSourceEconomics/estimagic).
78. J.-L. R. Stevens, P. Rudiger, J. Bednar, presented at the Proceedings of the 14th Python in Science Conference, pp. 61–69.
79. J. D. Hunter, Matplotlib: A 2D graphics environment. *Computing in science & engineering* **9**, 90–95 (2007).
80. S. K. Lam, A. Pitrou, S. Seibert, presented at the Proceedings of the Second Workshop on the LLVM Compiler Infrastructure in HPC, pp. 1–6.

81. C. R. Harris, K. J. Millman, S. J. van der Walt, R. Gommers, P. Virtanen, D. Cournapeau, E. Wieser, J. Taylor, S. Berg, N. J. Smith, R. Kern, M. Picus, S. Hoyer, M. H. van Kerkwijk, M. Brett, A. Haldane, J. F. del Río, M. Wiebe, P. Peterson, P. Gérard-Marchant, K. Sheppard, T. Reddy, W. Weckesser, H. Abbasi, C. Gohlke, T. E. Oliphant, Array programming with NumPy. *Nature* **585**, 357–362, (https://doi.org/10.1038/s41586-020-2649-2) (2020).

82. The pandas development team, *pandas-dev/pandas: Pandas*, version latest, 2020, (https://doi.org/10.5281/zenodo.3509134).

83. W. McKinney, presented at the Proceedings of the 9th Python in Science Conference, ed. by S. van der Walt, J. Millman, pp. 56–61.

84. T. Raabe, "A Python tool for managing scientific workflows.," 2020, (https://github.com/pytask-dev/pytask).

85. G. Van Rossum, F. L. Drake Jr, *Python reference manual* (Centrum voor Wiskunde en Informatica Amsterdam, 1995).

86. P. Virtanen, R. Gommers, T. E. Oliphant, M. Haberland, T. Reddy, D. Cournapeau, E. Burovski, P. Peterson, W. Weckesser, J. Bright, *et al.*, SciPy 1.0: fundamental algorithms for scientific computing in Python. *Nature methods* **17**, 261–272 (2020).

87. M. L. Waskom, Seaborn: statistical data visualization. *Journal of Open Source Software* **6**, 3021 (2021).

88. P. C. Silva, P. V. Batista, H. S. Lima, M. A. Alves, F. G. Guimarães, R. C. Silva, COVID-ABS: An agent-based model of COVID-19 epidemic to simulate health and economic effects of social distancing interventions. *Chaos, Solitons & Fractals* **139**, 110088, (https://doi.org/10.1016/j.chaos.2020.110088) (2020).

89. E. Cuevas, An agent-based model to evaluate the COVID-19 transmission risks in facilities. *Computers in Biology and Medicine* **121**, 103827, (https://doi.org/10.1016/j.compbiomed.2020.103827) (2020).

90. R. Hinch, W. J. M. Probert, A. Nurtay, M. Kendall, C. Wymant, M. Hall, K. Lythgoe, A. Bulas Cruz, L. Zhao, A. Stewart, L. Ferretti, D. Montero, J. Warren, N. Mather, M. Abueg, N. Wu, O. Legat, K. Bentley, T. Mead, K. Van-Vuuren, D. Feldner-Busztin, T. Ristori, A. Finkelstein, D. G. Bonsall, L. Abeler-Dörner, C. Fraser, OpenABM-Covid19—An agent-based model for non-pharmaceutical interventions against COVID-19 including contact tracing. *PLOS Computational Biology* **17**, 1–26, (https://doi.org/10.1371/journal.pcbi.1009146) (2021).

91. A. Aleta, D. Martín-Corral, A. P. y Piontti, M. Ajelli, M. Litvinova, M. Chinazzi, N. E. Dean, M. E. Halloran, I. M. L. Jr, S. Merler, A. Pentland, A. Vespignani, E. Moro, Y. Moreno, Modelling the impact of testing, contact tracing and household quarantine on second waves of COVID-19. *Nature Human Behaviour* **4**, 964–971 (2020).

92. M. Abueg, R. Hinch, N. Wu, L. Liu, W. Probert, A. Wu, P. Eastham, Y. Shafi, M. Rosencrantz, M. Dikovsky, Z. Cheng, A. Nurtay, L. Abeler-Dörner, D. Bonsall, M. V. McConnell, S. O'Banion, C. Fraser, Modeling the effect of exposure notification and non-pharmaceutical interventions on COVID-19 transmission in Washington state. *npj Digital Medicine* **4** (2021).

93. M. Bicher, C. Rippinger, C. Urach, D. Brunmeir, U. Siebert, N. Popper, Evaluation of Contact-Tracing Policies against the Spread of SARS-CoV-2 in Austria: An Agent-Based Simulation. *Medical Decision Making*, 0272989X2110133 (2021).

94. A. Basurto, H. Dawid, P. Harting, J. Hepp, D. Kohlweyer, Economic and Epidemic Implications of Virus Containment Policies: Insights from Agent-Based Simulations. *SSRN Electronic Journal* (2020).

95. D. Delli Gatti, S. Reissl, ABC: An Agent Based Exploration of the Macroeconomic Effects of Covid-19. *CESifo Working Paper Series*, (https://papers.ssrn.com/sol3/papers.cfm?abstract_id=3748964#) (2020).

96. P. Mellacher, "COVID-Town: An Integrated Economic-Epidemiological Agent-Based Model," MPRA Paper 103661 (University Library of Munich, Germany, 2020), (https://ideas.repec.org/p/pra/mprapa/103661.html).

97. C. Avery, W. Bossert, A. Clark, G. Ellison, S. F. Ellison, An Economist's Guide to Epidemiology Models of Infectious Disease. *Journal of Economic Perspectives* **34**, 79–104, (https://www.aeaweb.org/articles?id=10.1257/jep.34.4.79) (2020).

98. I. E. Isphording, M. Lipfert, N. Pestel, Does re-opening schools contribute to the spread of SARS-CoV-2? Evidence from staggered summer breaks in Germany. *Journal of Public Economics* **198**, 104426 (2021).

99. J. Gabler, T. Raabe, K. Röhrl, H.-M. v. Gaudecker, "Die Bedeutung individuellen Verhaltens über den Jahreswechsel für die Weiterentwicklung der Covid-19-Pandemie in Deutschland," tech. rep. (Institute of Labor Economics (IZA), 2020), (https://www.iza.org/publications/s/99/die-bedeutung-individuellen-verhaltens-uber-den-jahreswechsel-fur-die-weiterentwicklung-der-covid-19-pandemie-in-deutschland).

100. L. Morawska, J. W. Tang, W. Bahnfleth, P. M. Bluyssen, A. Boerstra, G. Buonanno, J. Cao, S. Dancer, A. Floto, F. Franchimon, C. Haworth, J. Hogeling, C. Isaxon, J. L. Jimenez, J. Kurnitski, Y. Li, M. Loomans, G. Marks, L. C. Marr, L. Mazzarella, A. K. Melikov, S. Miller, D. K. Milton, W. Nazaroff, P. V. Nielsen, C. Noakes, J. Peccia, X. Querol, C. Sekhar, O. Seppänen, S.-i. Tanabe, R. Tellier, K. W. Tham, P. Wargocki, A. Wierzbicka, M. Yao, How can airborne transmission of COVID-19 indoors be minimised? *Environment International* **142**, 105832 (2020).

101. E. L. Anderson, P. Turnham, J. R. Griffin, C. C. Clarke, Consideration of the Aerosol Transmission for COVID-19 and Public Health. *Risk Analysis* **40**, 902–907 (2020).

102. J. R. Donsimoni, R. Glawion, B. Plachter, K. Wälde, Projecting the spread of COVID-19 for Germany. *German Economic Review* **21**, 181–216, (https://www.degruyter.com/view/journals/ger/21/2/article-p181.xml) (2020).

103. R. L. Smith, L. L. Gibson, P. P. Martinez, R. Ke, A. Mirza, M. Conte, N. Gallagher, A. Conte, L. Wang, R. Fredrickson, D. C. Edmonson, M. E. Baughman, K. K. Chiu, H. Choi, T. W. Jensen, K. R. Scardina, S. Bradley, S. L. Gloss, C. Reinhart, J. Yedetore, A. N. Owens, J. Broach, B. Barton, P. Lazar, D. Henness, T. Young, A. Dunnett, M. L. Robinson, H. H. Mostafa, A. Pekosz, Y. C. Manabe, W. J. Heetderks, D. D. McManus, C. B. Brooke, Longitudinal assessment of diagnostic test performance over the course of acute SARS-CoV-2 infection. *The Journal of Infectious Diseases*, jiab337, ISSN: 0022-1899, (https://doi.org/10.1093/infdis/jiab337) (2021).

104. A. K. Lindner, O. Nikolai, F. Kausch, M. Wintel, F. Hommes, M. Gertler, L. J. Krüger, M. Gaeddert, F. Tobian, F. Lainati, L. Köppel, J. Seybold, V. M. Corman, C. Drosten, J. Hofmann, J. A. Sacks, F. P. Mockenhaupt, C. M. Denkinger, Head-to-head comparison of SARS-CoV-2 antigen-detecting rapid test with self-collected nasal swab versus professional-collected nasopharyngeal swab. *European Respiratory Journal* **57**, 2003961 (2020).

105. N. Kronfeld-Schor, T. J. Stevenson, S. Nickbakhsh, E. S. Schernhammer, X. C. Dopico, T. Dayan, M. Martinez, B. Helm, Drivers of Infectious Disease Seasonality: Potential Implications for COVID-19. *Journal of Biological Rhythms* **36**, 35–54 (2021).

106. M. J. Kühn, D. Abele, T. Mitra, W. Koslow, M. Abedi, K. Rack, M. Siggel, S. Khailaie, M. Klitz, S. Binder, L. Spataro, J. Gilg, J. Kleinert, M. Häberle, L. Plötzke, C. D. Spinner, M. Stecher, X. X. Zhu, A. Basermann, M. Meyer-Hermann, Assessment of effective mitigation and prediction of the spread of SARS-CoV-2 in Germany using demographic information and spatial resolution. (2020).

107. C. Betsch, L. Wieler, M. Bosnjak, M. Ramharter, V. Stollorz, S. Omer, L. Korn, P. Sprengholz, L. Felgendreff, S. Eitze, *et al.*, Germany COVID-19 Snapshot Monitoring (COSMO Germany): Rapid Tests. (https://projekte.uni-erfurt.de/cosmo2020/web/topic/wissen-verhalten/80-schnelltests/#best%C3%A4tigung-von-schnelltests-durch-pcr-tests-stand-23.03.21) (2021).

108. M. Pavelka, K. Van-Zandvoort, S. Abbott, K. Sherratt, M. Majdan, P. Jarčuška, M. Krajčí, S. Flasche, S. Funk, The impact of population-wide rapid antigen testing on SARS-CoV-2 prevalence in Slovakia. *Science* **372**, 635–641 (2021).

109. U. Berger, C. Fritz, G. Kauermann, "CODAG Bericht Nr. 14: 2. Schulschließungen oder Schulöffnung mit Testpflicht? Epidemiologisch-statistische Aspekte sprechen für Schulöffnungen mit verpflichtenden Tests," tech. rep. (Ludwig-Maximilians-Universität München, 2021), (https://www.covid19.statistik.uni-muenchen.de/pdfs/codag_bericht_14.pdf).

110. H. Scheiblauer, A. Filomena, A. Nitsche, A. Puyskens, V. M. Corman, C. Drosten, K. Zwirglmaier, C. Lange, P. Emmerich, M. Müller, O. Knauer, C. M. Nübling, Comparative sensitivity evaluation for 122 CE-marked rapid diagnostic tests for SARS-CoV-2 antigen, Germany, September 2020 to April 2021. *Eurosurveillance* **26**, (https://www.eurosurveillance.org/content/10.2807/1560-7917.ES.2021.26.44.2100441) (2021).

111. G. Cosentino, M. Bernard, J. Ambroise, J.-M. Giannoli, J. Guedj, F. Débarre, F. Blanquart, SARS-CoV-2 viral dynamics in infections with Alpha and Beta variants of concern in the French community. *Journal of Infection* **84**, 94–118, (https://doi.org/10.1016/j.jinf.2021.07.031) (2022).

112. S. W. X. Ong, C. J. Chiew, L. W. Ang, T.-M. Mak, L. Cui, M. P. H. S. Toh, Y. D. Lim, P. H. Lee, T. H. Lee, P. Y. Chia, S. Maurer-Stroh, R. T. P. Lin, Y.-S. Leo, V. J. Lee, D. C. Lye, B. E. Young, Clinical and Virological Features of Severe Acute Respiratory Syndrome Coronavirus 2 (SARS-CoV-2) Variants of Concern: A Retrospective Cohort Study Comparing B.1.1.7 (Alpha), B.1.351 (Beta), and B.1.617.2 (Delta). *Clinical Infectious Diseases*, ciab721, ISSN: 1058-4838, (https://doi.org/10.1093/cid/ciab721) (2021).

113. C. Bonnet, S. Masse, H. Benamar, A.-M. Vilcu, M. Swital, T. Hanslik, S. van der Werf, X. Duval, F. Carrat, A. Falchi, T. Blanchon, Is the Alpha Variant of SARS-CoV-2 Associated with a Higher Viral Load than the Historical Strain in Saliva Samples in Patients with Mild to Moderate Symptoms? *Life* **12**, ISSN: 2075-1729, (https://www.mdpi.com/2075-1729/12/2/163) (2022).

114. S. Jang, J.-Y. Rhee, Y. M. Wi, B. K. Jung, Viral kinetics of SARS-CoV-2 over the preclinical, clinical, and postclinical period. *International Journal of Infectious Diseases* **102**, 561–565, (https://doi.org/10.1016/j.ijid.2020.10.099) (2021).

115. M. Zuin, V. Gentili, C. Cervellati, R. Rizzo, G. Zuliani, Viral Load Difference between Symptomatic and Asymptomatic COVID-19 Patients: Systematic Review and Meta-Analysis. *Infectious Disease Reports* **13**, 645–653, ISSN: 2036-7449, (https://www.mdpi.com/2036-7449/13/3/61) (2021).

116. L. C. Rosella, A. Agrawal, J. Gans, A. Goldfarb, S. Sennik, J. Stein, Large-scale implementation of rapid antigen testing system for COVID-19 in workplaces. *Science Advances* **8**, eabm3608, (https://www.science.org/doi/abs/10.1126/sciadv.abm3608) (2022).
